# Heterospecific Neighbor Plants Impact Root Microbiome Diversity and Molecular Function of Root Fungi

**DOI:** 10.3389/fmicb.2021.680267

**Published:** 2021-11-04

**Authors:** Hui-Ling Liao, Gregory Bonito, Khalid Hameed, Steven H. Wu, Ko-Hsuan Chen, Jesse Labbé, Christopher W. Schadt, Gerald A. Tuskan, Francis Martin, Alan Kuo, Kerrie Barry, Igor V. Grigoriev, Rytas Vilgalys

**Affiliations:** ^1^North Florida Research and Education Center, University of Florida, Quincy, FL, United States; ^2^Department of Biology, Duke University, Durham, NC, United States; ^3^Department of Plant, Soil and Microbial Sciences, Michigan State University, East Lansing, MI, United States; ^4^Department of Agronomy, National Taiwan University, Taipei, Taiwan; ^5^Biodiversity Research Center, Academia Sinica, Taipei, Taiwan; ^6^Biosciences Division, Oak Ridge National Laboratory, Oak Ridge, TN, United States; ^7^Invaio Sciences, Cambridge, MA, United States; ^8^University of Lorraine, INRAE, UMR Interactions Arbres/Microorganismes, Champenoux, France; ^9^U.S. Department of Energy Joint Genome Institute, Lawrence Berkeley National Laboratory, Berkeley, CA, United States; ^10^Department of Plant and Microbial Biology, University of California, Berkeley, Berkeley, CA, United States

**Keywords:** microbiome, common mycorrhizal network (CMN), *Suillus*, metatranscriptomics, wood wide web, ectomycorrhizal fungi

## Abstract

Within the forest community, competition and facilitation between adjacent-growing conspecific and heterospecific plants are mediated by interactions involving common mycorrhizal networks. The ability of plants to alter their neighbor’s microbiome is well documented, but the molecular biology of plant-fungal interactions during competition and facilitation has not been previously examined. We used a common soil-plant bioassay experiment to study molecular plant-microbial interactions among rhizosphere communities associated with *Pinus taeda* (native host) and *Populus trichocarpa* (non-native host). Gene expression of interacting fungal and bacterial rhizosphere communities was compared among three plant-pairs: *Populus* growing with *Populus*, *Populus* with *Pinus*, and *Pinus* with *Pinus*. Our results demonstrate that heterospecific plant partners affect the assembly of root microbiomes, including the changes in the structure of host specific community. Comparative metatranscriptomics reveals that several species of ectomycorrhizal fungi (EMF) and saprotrophic fungi exhibit different patterns of functional and regulatory gene expression with these two plant hosts. Heterospecific plants affect the transcriptional expression pattern of EMF host-specialists (e.g., *Pinus*-associated *Suillus* spp.) on both plant species, mainly including the genes involved in the transportation of amino acids, carbohydrates, and inorganic ions. Alteration of root microbiome by neighboring plants may help regulate basic plant physiological processes via modulation of molecular functions in the root microbiome.

## Introduction

Temperate and boreal forest communities are often composed of a mix of plant species. These forest plants may release up to 40% of their photo-assimilated carbon into the soil, where it is dynamically transferred, along with soil nutrients, among neighboring plants through common mycorrhizal networks (CMNs) or “wood wide web” (Simard et al., 1997, 2012; Bais et al., 2006; Klein et al., 2016; Yin et al., 2018) and rhizosphere activities, which include rhizodeposit-mediated rhizosphere priming. As such, root symbionts play key roles in these belowground processes.

Rhizosphere priming is driven by root activities. The plant inter-species interactions that shape the allocation of plant carbon and chemical diversity of exudates may lead to restructuring of microbial communities and changes in the activities and function of rhizosphere microbes (el Zahar Haichar et al., 2008; Dijkstra et al., 2013; Pausch et al., 2013). These microbes can further mediate plant nutrient allocation and soil nutrient cycling by regulating rhizosphere priming (rhizosphere decomposition). CMNs is another critical strategy used by fungal symbionts to sustain soil microbial biomass, as well as to manipulate heterospecific nutrition networks (Dickie et al., 2002; Nara, 2006; Teste and Simard, 2008; van der Heijden and Horton, 2009). Through resource exchange facilitated by mycelial networks, some soil fungi have been shown to affect the establishment and survival of conspecific as well as heterospecific neighboring plants. For example, mycoheterotrophic plant populations were established through the support of neighboring photosynthetic plants (Bjorkman, 1960; Selosse et al., 2006; Smith and Read, 2008; Dighton, 2009). Douglas-fir and *Pinus* trees utilize ectomycorrhizal CMNs to shunt nutrients from overstory plant hosts to the nutrient-demanders (Aučina et al., 2011; Gorzelak et al., 2015; Song et al., 2015). Here, ectomycorrhizal fungi (EMF) in particular have an important role in mediating plant interactions via the CMNs (Kadowaki et al., 2018). The rhizosphere priming and the biochemical reactions and nutrient transferring within CMNs are regulated by many factors including individual plant-microbial interactions, host compatibility, competition, and facilitation among plants and soil microbes (Fellbaum et al., 2014; Bücking et al., 2016; Weremijewicz et al., 2016). Complex plant-soil feedbacks involving rhizosphere priming and CMNs have the potential to alter community composition and affect nutrient allocation among plant species.

Many microbes in the rhizosphere that interact with plants show little host specificity, thus, are called host-generalists. Host-generalist interactions have the potential to improve nutrient uptake for diverse plant species. In this case, the allocation of nutrients through the rhizosphere may largely depend upon the nutrient demand of individual plant species rather than host specificity (Fellbaum et al., 2014; Weremijewicz et al., 2016). However, some mutualistic microbes of plants exhibit various degrees of host-specificity toward different plant hosts. Examples of host-specific interactions are known for many species of EMF (Molina, 1992; Liao et al., 2016), some species of arbuscular mycorrhizal fungi (AMF) (Helgason et al., 2002, 2007), and nitrogen-fixing bacteria such as *Rhizobium* and *Frankia* (Andronov et al., 2015; Wang et al., 2018). These symbionts dominate the habitats of diverse plant species. Yet little is known on how hyphae formed by the mycorrhizal fungi with narrow host ranges (host-specialists) interact with non-host plant species grown nearby, or which functional genes are involved in such interactions? Addressing this knowledge gap is important to identify mechanisms that specialists apply to regulate nutrient fluxes between their plant host and heterospecific plant neighbors. After all, the ability of these generalists and specialists to mediate resource exchanges between plant hosts is critical in regulating downstream functional systems involving nutrient exchange, carbon partitioning, and growth. An understanding of the influence of a plant’s microbiome on that of its plant neighbor also has implications for ecological models scaling-up from whole plant to ecosystem level.

Mycorrhizal fungi (EMF and AMF) are the most dominant fungal symbionts of tree species (Robinson et al., 1997; Treseder and Cross, 2006; Futai et al., 2008). For example, all *Pinus* and *Populus* species are dependent on EMF, which are responsible for the substantial components of C/N fluxes in forest ecosystems (Molina, 1994; Liu et al., 2020). In addition to mycorrhizal fungi, the microbiome of a plant may also affect of diverse non-mycorrhizal microbes living in roots of its neighboring plants (e.g., root endophytes) (Khare et al., 2018). Previous molecular studies have shown that co-occurring plant hosts often harbor different communities of fungal endophytes (Morris et al., 2008; Tedersoo et al., 2008; Bonito et al., 2014), which implies that host-specificity may help to drive rhizosphere diversity, including fungal taxa with the ability to develop common mycelium networks. Overall, the growing body of research suggests that plant hosts are able to select and regulate the composition of their microbiome (Jones et al., 2019). Relatively few studies, however, have examined the effect of multiple plant species on functionality of soil microbiomes including CMNs associated fungi (Kadowaki et al., 2018; Compant et al., 2019).

Mechanisms governing how root microorganisms manipulate plant development have mostly been studied in simplified systems where a single strain of fungus or bacteria is paired with a single plant (Felten et al., 2009; Ortíz-Castro et al., 2009; Plett et al., 2011; Verbon and Liberman, 2016). However, metatranscriptomics offers the potential to study genome-wide expression of complex communities. In this study, we grew pairs of *Populus* and *Pinus* species in heterospecific and conspecific combinations to assess the influence of host’s microbiome on that of its plant neighbors. We hypothesized that heterospecific neighbor plants would alter each other’s root microbiome structure (especially mycorrhizal fungi) and gene expression, impacting the biomass of plant partners. In this study, we define root microbiome (root bacteria and root fungi) as the microbes living in or around the roots. We characterized gene expression of the whole root microbial community using metatranscriptomics to reveal molecular mechanisms governing the selection of fungal microorganisms and plant growth promotion. Within the same soil, different species of plants have been reported to harbor different communities of microorganisms (Viebahn et al., 2005; Garbeva et al., 2008). Here, we asked whether plant species exert selective effects on root microorganisms, and whether heterospecific neighboring plants alter the composition, structure, and molecular function (gene expression) of root microbial communities, particularly for the functional genes involved in nutrient transportation. To our knowledge, this is the first paper to investigate neighbor effects on the phylogenetic and functional diversity of plant microbiome at the metatranscriptomic level.

## Materials and Methods

### Plant Bioassay Experiment and Sample Collection

**FIGURE 1:**
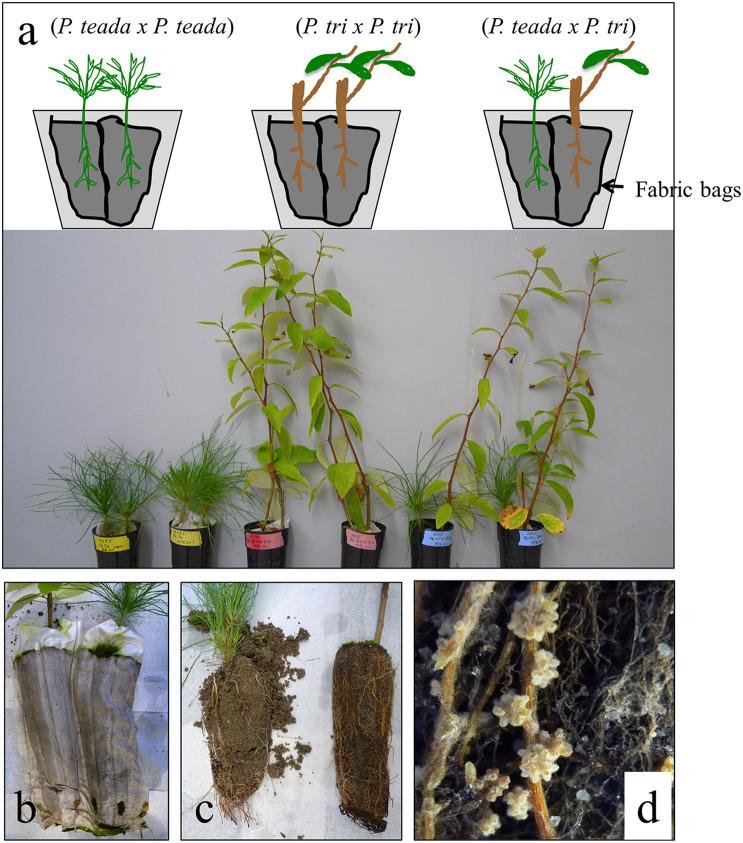
Heterospecific plant bioassay experimental setup. **(a)** Young plants of *Pinus taeda* and *Populus trichocarpa* BESC4 growing in different combination: *P. taeda* × *P. taeda*, *P. trichocarpa* × *P. trichocarpa*, and *P. taeda* × *P. trichocarpa*. **(b,c)** The roots of two plants grown in the same pot were separated using synthetic fabric “socks” to prevent direct contact between roots of each plant; **(d)** Ectomycorrhizal roots observed on *P. taeda* but not on *P. trichocarpa* (Supplementary Table 1). *P. tri* = *P. trichocarpa*.

**FIGURE 2:**
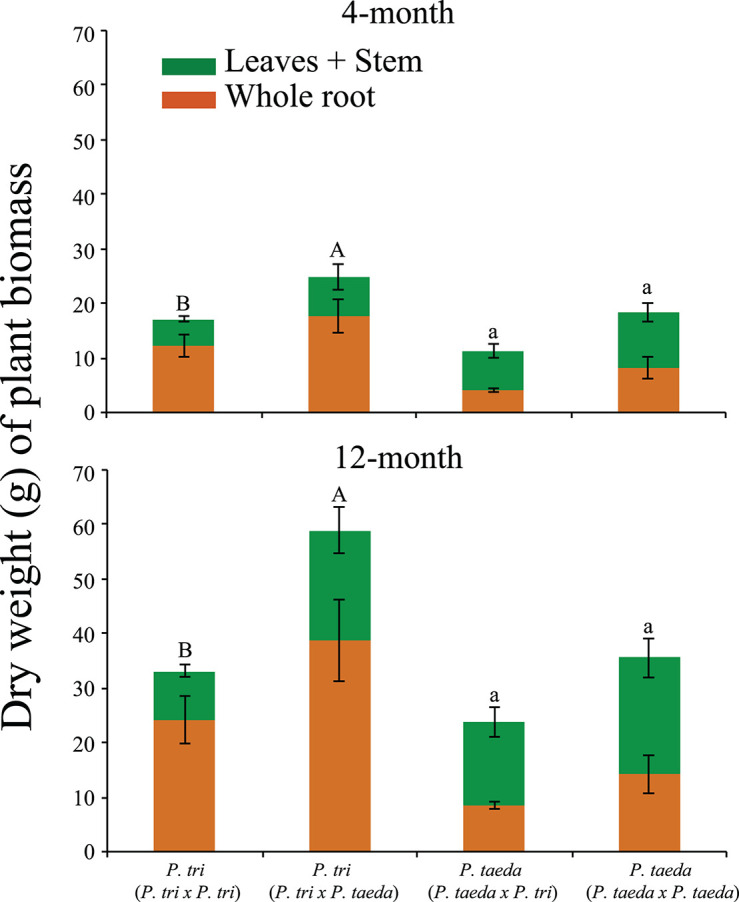
Plant biomass (dry weight and standard deviation) of aboveground plant (above) and root tissue biomass (below) for seedling grown under different plant combinations: *Populus trichocarpa* grown with *P. trichocarpa* (*P. trichocarpa* × *P. trichocarpa*), *P. trichocarpa* with *Pinus taeda* (*P. trichocarpa* × *P. taeda*), *P. taeda* with *P. trichocarpa* (*P. taeda* × *P. trichocarpa*) and *P. taeda* with *P. taeda*. Significant groupings determined by Tukey test for whole plant biomass for the individual plant species (*P* < 0.05, *n* ≥ 4). Means are marked by the same letter were not significantly different (Uppercase for *P. trichocarpa*; Lowercase for *P. taeda*). *P. tri* = *P. trichocarpa*.

To study *in situ* interactions between soil microorganisms with native (*Pinus taeda*) vs. non-native (*Populus trichocarpa*) hosts, a plant bioassay experiment was performed with fresh soils collected from a *P. taeda* forest site (Duke forest, Durham NC) in October 2014. Around 30 Kg soils (3–5 cm organic horizons, followed by 3 cm mineral horizons) were collected with a spade shovel. At the time of soil collection, the average temperature in these forest soils was 20°C. Collected soils were mixed with sterile sand [soil:sand = 3:7 (w/w)] and plants were grown with a partner in three combinations: (1) *Populus* × *Populus*, (2) *Populus* × *Pinus*, and (3) *Pinus* × *Pinus* (Figure 1). The growing conditions of plant materials prior to plant bioassay are described in Supporting Information Text A1. For each combination, 700 g of soil-sand mix was used to fill a 35-cm depth pot (cell volume, 983 ml). At least eight biological replicates were conducted for each treatment. An anti-static fabric bag with a mesh size of 60 um was used to physically segregate the roots of one plant from the other while allowing for microbial traffic between plants. This design allowed us to collect the microbial RNA and detect the expression of microbial genes from the roots of individual plant species. Each pot contained two bags, each of which contained 350 g of soil and one plant. Plants were grown in a growth chamber at 25°C, 80% humidity and fluorescent light at 200 μmol m^–2^ s^–1^ for 12 h per day. Whole plants were harvested at two time points, 4 months (*n* = 4) and 12 months (*n* = 4). At these times, plant dry weight biomass and mycorrhization levels were examined (Figure 2). EMF mycorrhization rates of *P. taeda* and *P. trichocarpa* roots were examined under a dissecting microscope. EMF mycorrhization rate was quantified by counting the% of mycorrhizal root tips for a total of 200 root tips for an individual sample. For plant dry weight measurement, the whole plants were washed with sterile DDI water, wiped with the sterile towel and then oven-dried for 72 h at 60°C, cooled to room temperature, separated the aboveground and belowground tissues, and then weighed.

Metatranscriptomic data was generated for samples taken at the 4-month time period (*n* ≥ 3 for biological replicates of each plant combinations–details below).

To sample roots, tertiary fine roots and root tips of *P. trichocarpa* and *P. taeda* were removed with forceps. Adhered soil was removed by shaking and forceps. Roots were then washed in sterile deionized water for <10 s to remove adhering soil particles. Distilled water was used to wash the roots, which were blotted dry on a clean paper towel then immediately frozen by liquid N_2_ and stored at −80°C until DNA/RNA extraction. The workflow for sampling and experimental design are summarized in Supplementary Figure 1.

### The Statistical Analysis for Plant Dry Weight and Mycorrhization Rate of Ectomycorrhizal Fungi

The means that were presented with bars (Figure 2) and in the table (Supplementary Table 1) showing the standard deviation of the mean. The significant difference between means was examined at *P* ≤ 0.05 using ANOVA followed by Tukey’s range test (*n* = 4).

### Metatranscriptomics and DNA/cDNA Amplicon Sequencing for Root Sample

**FIGURE 3:**
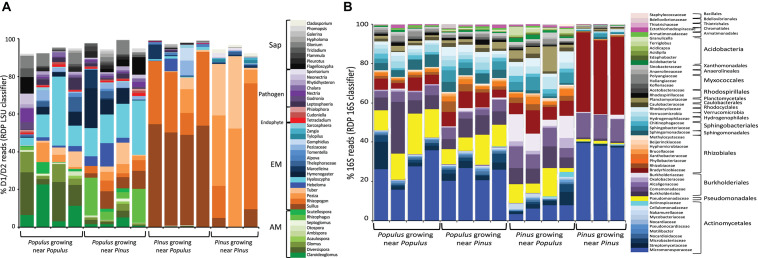
Relative transcriptomic abundances of fungal **(A)** and bacterial **(B)** taxa present in root samples determined by the relative proportion of rRNA LSU D1/D2 reads **(A)**, or 16S rRNA reads **(B)** recovered from roots of individual plants. The color key indicates fungal taxa grouped at genus level (RDP classifier with >60% bootstrap support). Reads belonging to unknown taxa and taxa with <0.5% reads were not included. Computational workflows for data analyses are shown in Supporting Information Text A2 and Supplementary Figure 2.

**FIGURE 4:**
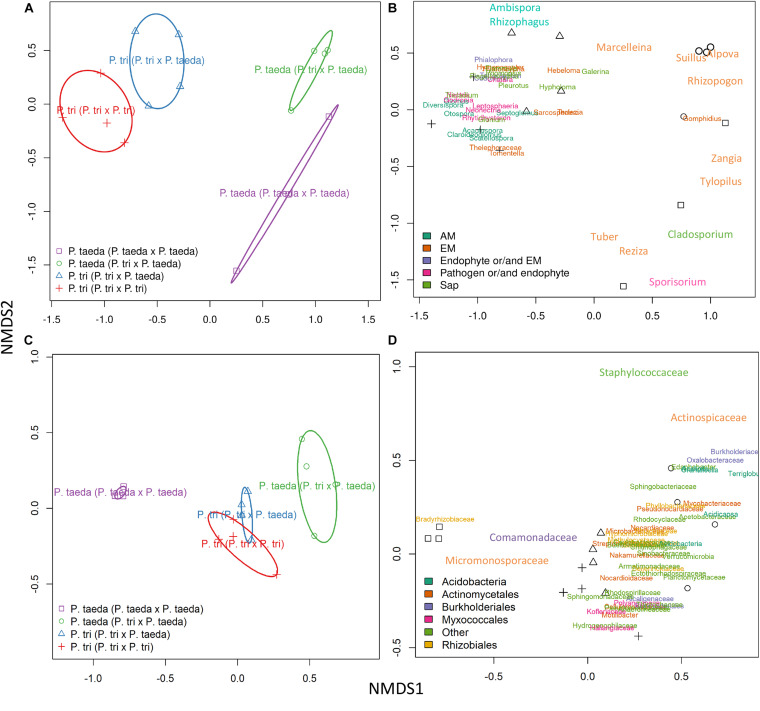
Non-metric multidimensional scaling plots showing the taxonomic composition of different plant host root microbiomes associated with same vs. different neighboring plant species. PERMANOVA was used to determine the changes of fungal **(A,B)** and bacterial **(C,D)** taxa associated with neighboring plant species (Supplementary Table 3 and Supplementary Datasets 2B,D). AMF, arbuscular mycorrhizal fungi; EMF, ectomycorrhizal fungi; Sap, Saprotrophic fungi. The pairwise comparisons were applied to each microbial taxon (Supplementary Datasets 2B,D). *P. tri* = *P. trichocarpa*.

DNA and RNA of root samples were co-extracted and purified according to Liao et al. (2014). RNA quantification, qualification and cDNA library construction for RNA-seq (125 PE, HiSeq 2000, Illumina, San Diego, CA, United States) was performed as previously described (Liao et al., 2014). Fifteen samples were sequenced on 1.3-lanes of Illumina HiSeq generating ∼60 Gb of data. Raw reads were deposited in the NCBI Short Read Archive (accession SRP156890). Bioinformatic pipelines for data assembly, quality filtering, annotation and comparison are described in Supporting Information Text A2. Briefly, ∼440 millions of qualified reads with a quality score >20 (FastQC analysis) were used for downstream analysis (Supplementary Figure 2, Step S1). The divergent domains of rRNA reads [fungal ribosomal RNA large submit rRNA D1/D2 region (LSU D1/D2) and bacterial rRNA 16S] were recovered from RNA-seq and were used to examine the taxonomic composition of fungi and bacteria, respectively (Supplementary Figure 2, Steps S2–S5). A total of 29,342 D1/D2 sequences downloaded from RDP classifier (Cole et al., 2014; Deshpande et al., 2016), and NCBI were used as the references. Reference-based mapping approach was used to quantify and normalize the reads belonging to fungal LSU D1/D2 and bacterial 16S using Bowtie2, SAMtools, and Picard (Li et al., 2009). The alignment options were described in Supporting Information Text A2. *P. trichocarpa* and *P. taeda* databases were performed to filter out D1/D2 reads belonging to plastid and mitochondrial rRNA genes. To generate Figures 3A,B, the percentage of reads was calculated according to the ratio of reads from individual taxa vs. total rRNA reads obtained from the individual samples (Supplementary Figure 2, Step 4). The ecological function of each fungal group was assigned according to FUNGuild (Nguyen et al., 2016). Differences in community composition among the comparisons were tested using permutational multivariate analysis of variance (PERMANOVA). Results for PERMANOVA were corrected for multiple comparison using false discovery rate (FDR). *P*-value were calculated based on pseudo-F statistics, and results with *P* ≤ 0.05 were considered as statistically significant (Results shown in Figures 4A–D; Supplementary Table 3; and Supplementary Datasets 2B,D). Both non-metric multidimensional scaling (NMDS) and PERMANOVA were performed using vegan package version 2.5.3 in R (3.5.1). The same RNA was used for amplicon sequence analysis targeting fungal LSU rDNA with the primers LROR and LR3 (Vilgalys and Hester, 1990). Details for amplicon sequencing are described in Supporting Information Text A3. To filter out plant reads, remaining unmapped reads (approximately 30-million; Supplementary Figure 2, Step S6) were mapped onto reference sequences of *P. trichocarpa* and *P. taeda* using TopHat and Bowtie2 packages (detailed see Supporting Information Text A2).

**FIGURE 5:**
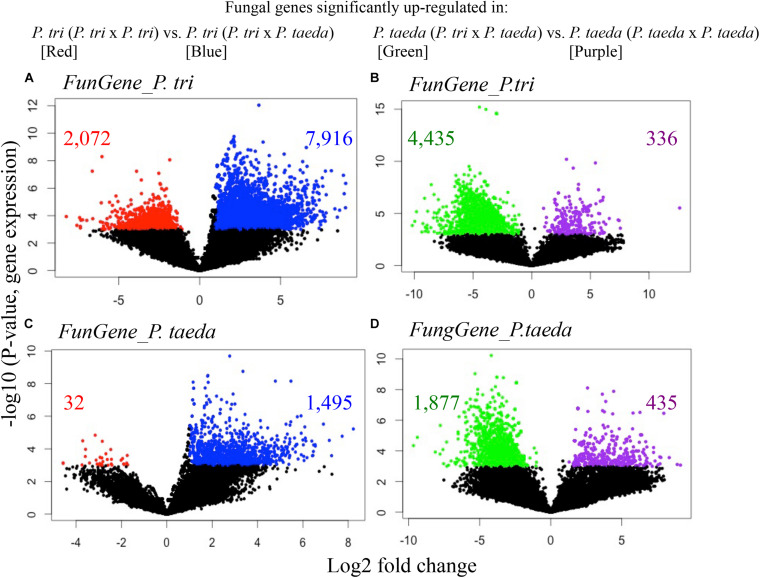
Volcano plots show the expression of fungal genes originally expressed in *Populus trichocarpa* roots (*FunGene_P. tri*, **A,B**) in *Pinus taeda* roots (*FunGene_P. taeda*, **C,D**) in response to neighbor plant species. **(A)** *FunGene_P. tri* on *P. trichocarpa* roots that were up-regulated (in blue) or down-regulated (in red) when grown with *P. taeda* compared to with *P. trichocarpa*; **(B)** *FunGene_P. tri* on *P. taeda* roots that were up-regulated (in green) or down-regulated (in purple) when *P. taeda* was grown with *P. trichocarpa* compared to with *P. taeda.* **(C)** *FunGene_P. taeda* on *P. trichocarpa* roots that were up-regulated (in blue) or down-regulated (in red) when grown with *P. taeda* compared to when grown with *P. trichocarpa*; **(D)** *FunGene_P. taeda* on *P. taeda* roots that were up-regulated (in green) or down-regulated (in purple) when *P. taeda* was grown with *P. trichocarpa* compared to when grown with *P. taeda.* Data of loading gene factors were generated using coordinate scales on the left (log10 of expression rate) and the bottom (mean of log2-fold changes). Cross-comparative expression of the genes was analyzed using *t*-test to compare the fungal genes of the roots from a plant species grown with conspecific vs. heterospecific neighbors (*n* ≥ 3; Wilcox.Test *P* < 0.01; FDR < 0.05; fold changes ≥ 2). The total counts of genes detected and significant changes in gene counts in response to neighbor effects are listed in Supplementary Dataset 3.

**FIGURE 6:**
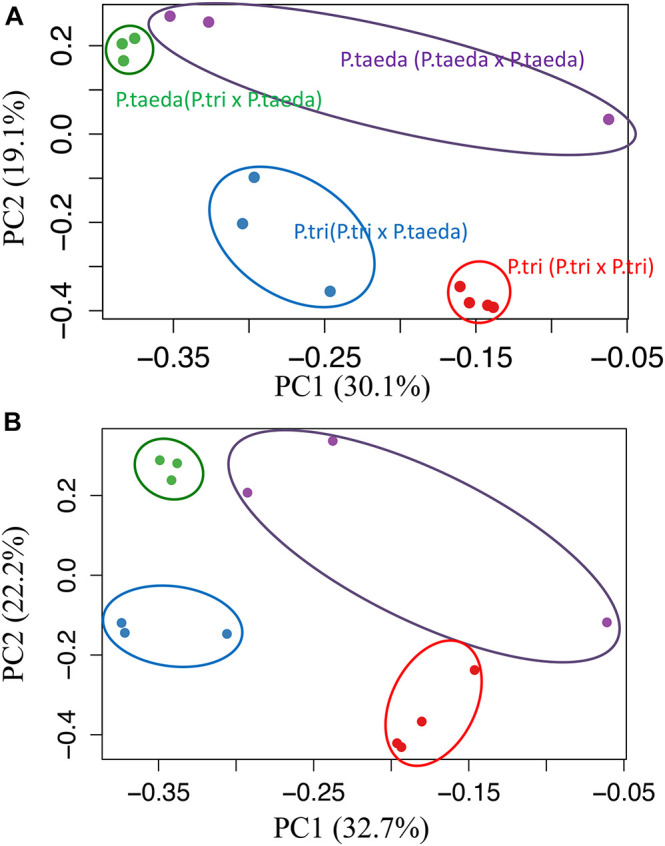
Shifts in gene expression patterns of root microbiomes associated with plant species. The principal component analysis of expression patterns for **(A)** “FUNGAL GENES originally expressed in *Populus trichocarpa* root (*FunGene_P. tri*)” (∼482K contigs), and **(B)** “FUNGAL GENES originally expressed in *Pinus taeda* root (*FunGene_P. taeda*)” (∼155K contigs). Color-filled circles show loadings of whole gene groups for root samples across different host combinations [*P. trichocarpa* (*P. trichocarpa* × *P. trichocarpa*) in red; *P. trichocarpa* (*P. trichocarpa* × *P. taeda*) in Blue; *P. taeda* (*P. trichocarpa* × *P. taeda*) in green; *P. taeda* (*P. taeda* × *P. taeda*) in purple; *n* ≥ 3). The distance between points approximates gene expression pattern differences among the samples. The percentage of variances were indicated in the parentheses. Root-microbiome genes were sorted using *de novo* assembly. Computational workflows for data analyses are shown in Supporting Information Text A2, Supplementary Figures 2, 3, and Supplementary Dataset 3.

**FIGURE 7:**
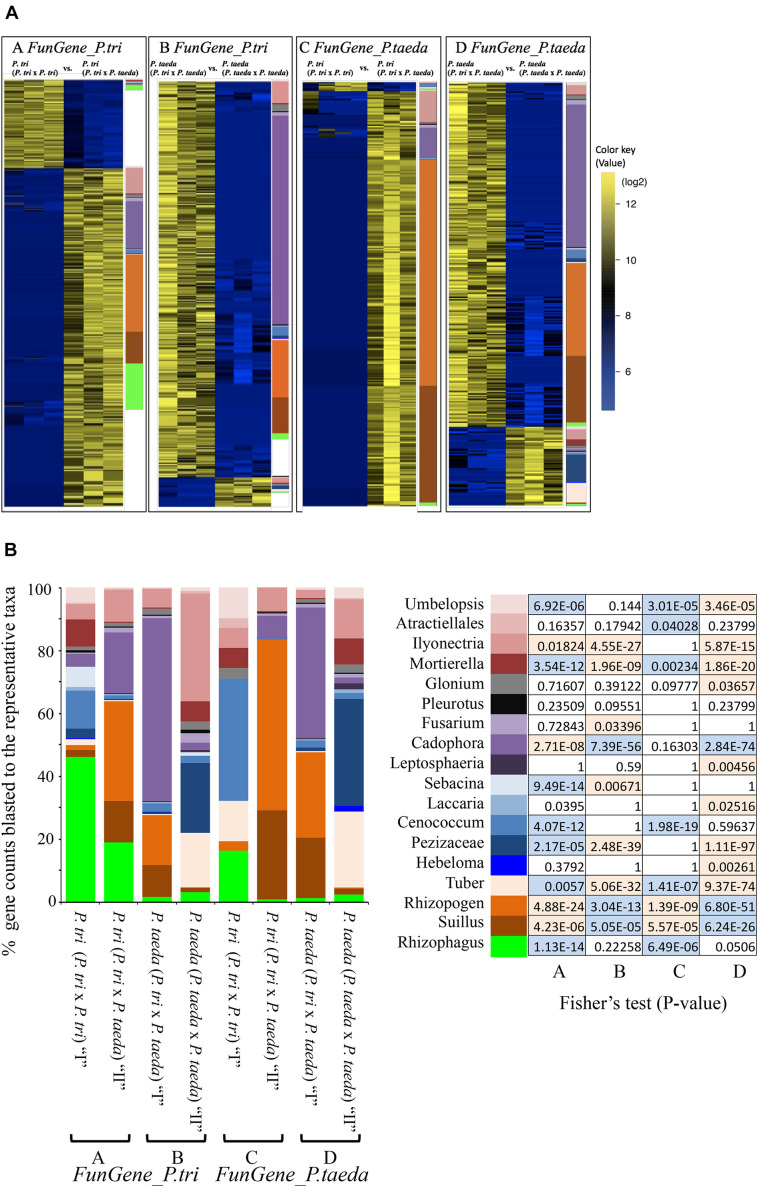
The changes in expression of two sets of fungal genes (*FunGene_P. tri*–A and B and *FunGene_P. taeda*–C and D) underlying different plant combinations. **(A)** Heat maps showing relative expression patterns of the *FunGene_P. tri* and *FunGene_P. taeda* that were up- or down-regulated in response to the plant traits (data were extracted from the color dots in Figure 4). The color bar on the right side of each heatmap [as well as in (B)] represents the numbers of genes assigned to predicted fungal taxa through blast (BlastX) to the fungal protein databases (method in Supporting Information Text A2). Data that were used to generate heatmaps are presented in Supplementary Dataset 3. Significant changes of gene expression were clustered according to ecological functions of fungal taxa, including AMF, EMF, Pathogens, Saprotrophs, and endophytes (>2-fold changes; FDR < 0.05). The color key represents RPKM normalized log2 transformed counts of the genes. Wilcoxon signed-rank test (Bauer, 1972) was applied to filter data. **(B)** Fisher’s test was used to identify enrichment of genes from the individual taxa underlying 2 × 2 matrix. Boxes in blue showed the genes of the individual taxa were enriched in “I” vs. “II.” Boxes in orange showed the genes of the individual taxa were enriched in “II” vs. “I” (*P* < 0.05).

To determine the genes belonging to root fungi that were differentially expressed across the three combinations of plant partners, we applied a combination of genome-based mapping and *de novo* approaches to sort out RNA-seq reads belonging to key functional genes of fungi (Supplementary Figure 2). Firstly, we removed the reads belonging to rRNA or plant genes. The *de novo* approach was then applied to assemble the poly-A selected “not-plant reads” (fungal reads). We then pooled and assembled *de novo* reads into contigs to serve as references (Supplementary Figure 3) to identify expression patterns of fungal genes from individual root samples (Figure 5–7). Using this approach we were able to sort out gene candidates for “Fungal genes originally expressed in *P. trichocarpa* roots” (*FunGene_P. tri*) and “Fungal genes originally expressed in *P. taeda* root” (*FunGene_P. taeda*). Here, the terms “*FunGene_P. tri*” and “*FunGene_P. taeda*” references represent the assembled reads that were originally collected from *P. trichocarpa* and *P. taeda* root, respectively. Since most of the pine root tips were heavily colonized by EMF (Supplementary Table 1), we expected large proportion of contigs assembled to “*FunGene_P. taeda*” belong to EMF genes. Compared to “*FunGene_P. taeda*,” the contigs of “*FunGene_P. tri*” reference may contain fungal genes with higher diverse ecological function (e.g., including AMF, EMF, and endophytes). Comparative transcriptomics were furthered applied for the normalized mapped-reads with the DESeq package (Figures 5, 6; Anders and Huber, 2010). Here, the genes of *FunGene_P. tri* and *FunGene_P. taeda* were assigned to the function (KOG) of closely relative fungal taxa using BlastX. The annotated protein sequences from 24 fungal genomes (Supporting Information Text A2_4) were used as references for mapping transcripts. These references were selected based upon their phylogenetic association with dominant fungal taxa detected in our dataset (see Figure 3A) [e.g., *Rhizophagus*, *Suillus*, *Rhizopogon*, *Tuber*, *Hebeloma*, Pezizaceae (*Terfezia*), *Leptosphaeria*, *Fusarium*, *Pleurotus*, and *Glonium*], the dominant soil/root microbiomes that were presented in the *Populus* sites (e.g., *Cenococcum*, *Laccaria*, *Ilyonectria*, *Mortierella*, *Atractiella*, *Umbelopsis*, and a dark septate endophyte, *Cadophora*) (Bonito et al., 2014). The sequences of the functional genes were assigned to the predicted trophic designations (e.g., genes belong to AMF, EMF) (Supplementary Dataset 4). Here, the classification of a gene to the taxa relied on the sequence that was assigned to a protein reference taxonomy via BlastX (Threshold:% ID > 50, *e* ≤ 10^–8^, alignment length > 50). The representative ecological group of each assigned gene was further designated based on the predicted ecological function of the assigned reference. The R-scripts used for this study can be accessed through: https://github.com/NFREC-Liao-Lab/Neighbor_plant_microbiome_RNA.

## Results and Discussion

### The Neighbor Effects on *P. taeda* and *P. trichocarpa*, Including Plant Biomass and Ectomycorrhizal Fungi Associated Mycorrhization

Plant bioassay experiments were conducted with *Pinus* and *Populus* plants grown together in pairwise combinations in a common soil to study neighbor effects on plant growth, root community and gene expression (Figure 1a). When *P. trichocarpa* was grown alongside *P. taeda*, there was a significant increase in *P. trichocarpa* biomass, compared to when *P. trichocarpa* was grown with a conspecific neighbor (*P* < 0.05, Figure 2). Overall, the growth differences of heterospecific plant species shown in Figure 2 may be mediated by microbial feedbacks (van der Heijden and Horton, 2009), the direct intraspecific plant-plant competition for resources (Craine and Dybzinski, 2013), or the interactive effects from both factors. While *P. trichocarpa* biomass increased significantly when grown with *P. taeda*, *P. taeda* biomass decreased, but not significantly, when grown with a *P. trichocarpa* neighbor (*P* > 0.05, Figure 2).

We hypothesized that heterospecific neighbor plants would alter the root community in both plant species, especially *Pinus* associated EMF, and their gene expression. For example, we expected some of the EMF specialists from *Pinus* soil may not be able to develop the full compatibility (e.g., mycorrhizae formation) with non-host plant species, however, might still be able to communicate with non-hosts through molecular interactions (Liao et al., 2016). The measurements of the EMF mycorrhization (this section) and the community and gene expression of root microbiome (next sections) allow us to examine if neighboring plant may affect the distribution and activity of the key agents (e.g., EMF) that are responsible for plant nutrient adaptation.

As mentioned above, the root microbiome of *Pinus* is dominated by EMF, many of which show high host-specificity (Smith and Read, 1997; Liao et al., 2014; Vincenot and Selosse, 2017), while *P. trichocarpa* and other *Populus* species typically exhibit lower levels of EMF colonization and diversity (Khasa et al., 2002; Danielsen et al., 2013; Bonito et al., 2014; Liao et al., 2019). Consistent with previous studies, we observed a slower progression of mycorrhizal development on *Populus* root tips compared to that of *Pinus* (Supplementary Table 1). For instance, there were low numbers of EMF root tips on *Populus* cuttings during the first 4 months of growth. Previous studies found that it may take up to 2 years to have ∼50% of the *P. trichocarpa* root tips colonized by EMF (Baum et al., 2002; Danielsen et al., 2013). Over 80% root tips of the *Pinus* seedlings were colonized with well-formed ectomycorrhizae (Figure 1d and Supplementary Table 1). In contrast, less than 2% of *P. trichocarpa* root tips showed visible mycorrhizas. Of interest, growing *P. taeda* near *P. trichocarpa* did not induce ectomycorrhizas on *Populus* or change EMF mycorrhization rates of either plant species. The result implies that many *Pinus*-associated EMF may not be able to form mycorrhizae on *Populus* roots.

### Identification of Molecular Activities of Plant Root Microbiomes Through Metatranscriptomics

We employed metatranscriptomics to examine root tips of *P. taeda* and *P. trichocarpa* grown in combinations of heterospecific and conspecific plant bioassay treatments (Figure 1a). For *P. taeda*, the root metatranscriptome of *Pinus* grown in *Pinus* forest soil exhibited a fairly constant proportionality of reads belonging to fungi and *P. taeda*. The% reads mapped to fungi, *P. taeda*, and others (reads not mapped to genome references) were 24% (stdev. 3.2%), 27% (stdev. 1.9%), and 47%, respectively (Supplementary Dataset 1). In contrast, fewer fungal reads were recovered from *Populus* roots compared to *Pinus* root systems. On average, the% reads mapped to fungi, *Populus*, and others (reads not mapped to genome references) were 5% (stdev 0.4%), 59% (stdev 1.6%), and 37%, respectively. Excluding ribosomal RNA reads (see next section), 37% (from *Pinus* root samples) and 47% of the reads (from *Populus* root samples) did not map to fungal genomes or plant transcriptomic database. Some of these reads may belong to plant and fungi, however, were not mapped due to lower abundance of these genes or genome variation within a species. Some of the unmapped reads may belong to the fungi which have not been molecular characterized. These sequences were excluded from the analysis. For example, relatively few full genome sequences are publicly available to serve as the databases for AMF and EMF species, indicating that some of the excluded reads might belong to the unknown taxa of mycorrhizal fungi. The attempts to identify the expression pattern of mycorrhizal fungi and other root microbes can be limited by the limitation of current available genome databases.

A portion of reads belonging to rRNA were recovered from RNA-seq data using poly-A enrichment strategy for cDNA library construction. In average, 53,943 fungal LSU D1/D2 reads and 144,013 bacterial 16S rRNA reads were recovered from each sample (Supplementary Dataset 1). These recovered reads allowed us to estimate fungal community structure from each sample based the transcriptomic abundance of prokaryotic and eukaryotic rRNA (Liao et al., 2014). Since rRNA is one of the basic components of microbial cells, it is likely to be an ideal indicator for principal activity (e.g., protein synthesis) of fungi and bacteria. The average ratio of fungal: bacterial rRNA in root tissues was around 1.5:1 (Supplementary Table 2). Growing *Pinus* and *Populus* together resulted in an increase of fungal-to-bacterial rRNA ratio to 2:1 in both plant species (Supplementary Dataset 1). The ratio of fungi:bacteria rRNA reads and the number of fungal species per root sample increased in heterospecific treatments (Supplementary Table 2 and Figure 3). Previous study showed that higher fungal:bacterial ratio may lead to higher soil carbon storage and nitrogen allocation to plant species (Malik et al., 2016) and result in facilitating plant growth promotion. It is also possible that heterospecific plant species may enhance the complexity of root exudates or elicitors released in the shared soil ecosystem (Grayston et al., 1997). The greater chemical diversity of root exudates may positively correlate to the diversity of microbes recruited by the roots (Steinauer et al., 2016). Our data showed that the fungal/bacterial rRNA ratio quickly responded to the plant treatments (4-month after bioassay). This indicates that fungal-based pathways in the root increased quickly in heterospecific treatments and may therefore dominate nutrient regulation compared to bacterial-based pathways. This, however, does not necessarily result in the changes of plant biomass. Additional studies are needed to examine if the cost-benefit ratio of heterospecific plant species can be mediated through belowground fungal networks.

### The Differences of Community Structure and Relative Abundance of Root Microbiome in *Populus* vs. *Pinus*

The fungal and bacterial rRNA communities differed substantially between *Populus* vs. *Pinus* roots (PERMANOVA, *P* < 0.05) (Figures 3A, 4A,B and Supplementary Table 3). *Populus* roots were dominated by AMF, representing 36% of total fungal rRNA D1/D2 reads for the individual root samples, followed by EMF (26%). Fungal rRNAs belonging to putative saprotrophs (18%), pathogens (16%) and endophytic fungi (4%) were also consistently observed in *Populus* roots (Figure 3A). The most common fungal taxa of *Populus* roots include *Claroideoglomus* (AMF), *Diversispora* (AMF), *Hebeloma* (EMF), *Hyaloscypha* (EMF), *Peziza* (putative-EMF) and *Tuber* (EMF). Interestingly, *Populus* roots were dominated by a similar group of fungal taxa regardless of the presence of heterospecific neighboring plants. Previous studies where *P. trichocarpa* cuttings were grown individually also found that *Claroideoglomus*, *Diversispora*, *Glomus*, *Rhizophagus*, *Tuber*, *Hebeloma*, and *Hyaloscypha* were dominated in *Populus* roots independent of whether cuttings were grown in the soils collected from *Pinus* sites (Figure 3) or *Populus* sites (Liao et al., 2019), indicating these taxa may comprise part of the core mycobiome of *Populus*.

Consistent with a previous study (Liao et al., 2014), EMF accounted for ∼95% of total fungal rRNA D1/D2 reads obtained from *Pinus* roots. Two host-specific taxa *Suillus* and *Rhizopogon* were dominant on *Pinus* roots, and represented over 80% of fungal rRNA D1/D2 reads, implying that compared to other EMF taxa, *Suillus* and *Rhizopogon* may better adapt to young seedlings. It also implies their ability to facilitate seedling establishment in pine forests (Policelli et al., 2019). A small portion (<5% of total fungal rRNA D1/D2 reads) of transcriptomically active pathogenic (*Sporisorium*) and saprotrophic (*Cladosporium*) fungi were also detected in ectomycorrhizal roots of *Pinus*. Pairwise comparisons between *P. trichocarpa* (*P. trichocarpa* × *P. trichocarpa*) vs. *P. taeda* (*P. taeda* × *P. taeda*) suggested that *Populus* harbored a higher relative abundance of six AMF taxa (*Claroideoglomus*, *Diversispora*, *Glomus*, *Otospora*, *Septoglomus*, and *Scutellospora*), four EMF taxa (*Hyaloscypha*, *Hymenogaster*, Pezizaceae, and *Sarcosphaera*), and 12 other fungal taxa with endophytic, pathogenic or/and saprotrophic activities (*P* < 0.05, Supplementary Dataset 2B); *Pinus* were dominated by three fungal taxa: *Rhizopogon*, *Marcellenina*, and *Cladosporium*. Overall, the diversity of microbial taxa was distinct in the roots of different plant species, even when the plants were grown in the same soil type. Plant host species was shown to influence the colonization ability of specific EMF species. These results indicate that host preference plays a substantial role in the composition and rRNA-associated metabolic activities of EMF and some other specific taxa on *Populus* and *Pinus*.

On average, 0.5% of the total RNA-seq reads that belong to 16S rRNA from the individual root samples were recovered, accounting for 144K reads in average (Supplementary Dataset 1). A total of 196 bacterial families were detected in roots of *P. trichocarpa* and *P. taeda* (Figure 3B and Supplementary Dataset 2C). When grown with a conspecific neighbor, *P. trichocarpa* interacted with broad diversity of bacteria and were composed of 5 major orders and 25 major families. These included members of Actinomycetales (30% of total 16S reads), Pseudomonadales (14%), Burkholderiales (9%), Rhizobiales (8%), and Sphingomonadales (5%). Acidobacteria, previously reported to be reduced in the *Populus* root endosphere (Shakya et al., 2013), were represented by only <1% 16S reads. *P. taeda* were dominated by three families of bacteria including Micromonosporaceae (Actinomycetales, 38% of total 16S rRNA read), Comamonadaceae (Burkholderiales, 12%), and Bradyrhizobiaceae (Rhizobiales, 38%) (Figure 3B). These three bacterial families have been reported as common root-associated microbial groups in conifers (Nguyen and Bruns, 2015). Of the 51 top families detected in the root tissues, 28 taxa had significantly higher relative abundance in *P. trichocarpa* (*P. trichocarpa* × *P. trichocarpa*) vs. *P. taeda* (*P. taeda* × *P. taeda*), and only two families (Comamonadaceae and Bradyrhizobiaceae) showed higher relative abundance in *P. taeda* (*P. taeda* × *P. taeda*) (Supplementary Dataset 2D). Taken together, our results indicate that *P. trichocarpa* and *P. taeda* each develop a distinct root fungal and bacterial community, even when growing in the same soil.

### Re-assembly of Root Microbiome of *P. trichocarpa* Partnered With *P. taeda*

To study whether plant-neighbors can alter the composition of their root microbiome, we used fungal D1/D2 rRNA and bacterial 16S rRNA sequence data to compare the transcriptome composition patterns of microbial taxa along the root systems of three compositions: *P. trichocarpa* × *P. trichocarpa*; *P. trichocarpa* × *P. taeda*; *P. taeda* × *P. taeda*. Distinct patterns of fungal and bacterial community were detected on *P. trichocarpa* and *P. taeda* root tips when these plant species were grown together (Figures 3, 4 and Supplementary Table 3) (*P* < 0.05). The results verify that plant species can select for specific root microbiomes (in terms of maintaining high relative abundances of specific microbiome), even with heterospecific plants growing nearby. However, the community structures of certain fungal and bacterial taxa can be significantly affected by the species of neighboring plants (*P* < 0.05, Supplementary Tables 3B,D). For example, the relative abundance of some fungal taxa increased (*Suillus*, *Rhizopogon*) or decreased (15 taxa) when *P. trichocarpa* was grown with *P. taeda* compared to when *P. trichocarpa* was grown with a conspecific neighbor (*P* < 0.05; Supplementary Dataset 2B). Although *Pinus* roots were still dominated by their own associated EMF (e.g., *Suillus*, *Rhizopogon*) while growing with *P. trichocarpa*, a few *P. trichocarpa-*associated EMF (*Hebeloma*, *Hyaloscypha*, *Hymenogaster*, and *Sarcosphaera*), AMF (*Claroideoglomus*, *Diversispora*, and *Rhizophagus*) and endophytes (*Tetracladium* and *Chalara*) and other five taxa increased their relative abundance of rRNA on these *Pinus* roots (Figure 3A; *P* < 0.05, Supplementary Dataset 2B). These results imply that EMF hypha extending from a compatible host root (the mycorrhizal root) are able to reach and interact with heterospecific plant roots even when the second host appears to be incompatible based on morphological development (Liao et al., 2016). Also connected by CMNs, in this instance, there was no evidence of mycorrhizal development on roots of the incompatible *Populus* host (Supplementary Table 1). To verify the consistency between key dominant taxa detected in RNA-seq data compared to next generation amplicon sequencing data, cDNA synthetized from rRNA from the same RNA extraction were generated from a subset of samples to obtain full sequences of the LSU D1/D2 region (Supplementary Figure 4). Fungal composition detected by cDNA amplicon sequencing targeting rRNA revealed similar patterns as RNA-seq data. When growing near *Pinus*, the *Populus* samples harbored a greater amount of AMF and endophytes (Leotiomycetes) compared to *Pinus* roots. Boletales (particularly *Suillus* and *Rhizopogon*) were consistently abundant in *Pinus*, which dominate the *Pinus* rhizosphere as uncovered by RNA-seq data (Figure 3 and Supplementary Figure 4). Fungal reads of *Tuber* spp. (Pezizales) were abundant in RNA-seq data of *Pinus* but not in cDNA amplicon sequencing data. RNA-seq reads assigned to *Suillus* were examined by aligning to *Suillus* reference sequences (D1/D2 regions obtained from fruiting bodies). Results confirm that merged short reads could be aligned to *Suillus* reference sequences, mostly along the conserved area between D1 and D2 regions (Supplementary Figure 5).

Bacterial rRNA diversity also varied between plant hosts. Micromonosporaceae, Bradyrhizobiaceae, and Comamonadaceae were the dominant families in both *P. trichocarpa* and *P. taeda* roots regardless of neighbor plant species (Figure 3B). *P. trichocarpa* however harbored a relatively higher diversity of bacterial taxa, thus may have a greater responsibility for shaping the composition of bacterial activities on *P. taeda* (Figures 4C,D; *P* < 0.001, Supplementary Table 3C). The potential influences of EMF species on the selection of bacterial community within *Pinus* ectomycorrhizas have been suggested (Nguyen and Bruns, 2015; Marupakula et al., 2016). However, members of Actinomycetales, Burkholderiales, and Rhizobiales were also present on *Populus* roots that harbor distinct communities of fungi (Figure 3B). These results indicate there is lower specificity of these bacterial members (at order or family level) to specific hosts or taxa of root-associated fungi, including EMF.

### Plant Partners Are Responsible for Shaping Gene Expression of the Root Microbiomes

In order to study functional dynamics of microbiomes across multiple plant species, we conducted paired-comparisons for the expression patterns of fungal genes from individual root samples (Figures 5–7). Specifically, the expression of the gene candidates for “Fungal genes originally expressed in *P. trichocarpa* roots” (*FunGene_P. tri*) and “Fungal genes originally expressed in *P. taeda* root” (*FunGene_P. taeda*) were compared. Comparative transcriptomics was further analyzed for the normalized mapped-reads with the DESeq package (Figures 5, 6; Anders and Huber, 2010). Many genes from both gene groups (*FunGene_P. tri* and *FunGene_P. taeda*) were detected on *P. trichocarpa* and *P. taeda* roots (Figure 5). Expression patterns of fungal functional genes clustered primarily by plant species (Figure 6), indicating that the microbial shifts caused by heterospecific plant neighbors (Figure 4) could cause changes in microbial gene expression. For example, for both individual plant species gene expression patterns of fungal microbiomes in roots were altered when plants were grown with heterospecific hosts (Figures 5, 6). A total of 7,916 of *FunGene_P. tri* genes on *P. trichocarpa* root were significantly upregulated when growing nearby *P. taeda* compared to conspecific combination (*P. trichocarpa* × *P. trichocarpa*, 2,072 genes) (Figure 5A). The 1,187 of *FunGene_P. taeda* genes on *P. taeda* root were significantly upregulated when growing nearby *P. trichocarpa* compared to conspecific combination (*P. taeda* × *P. taeda*, 435 genes) (Figure 5D). Interestingly, the difference in composition of root microbiome in response to heterospecific treatment appeared greater for *P. taeda*, compared to *P. trichocarpa* (Figures 4A,C), and *P. taeda* roots harbored more fungal biomass (mostly EMF) than *P. trichocarpa* roots (Supplementary Table 1). However, the shift of gene expression pattern (Figures 5A,D) and total counts of upregulated genes (Figure 6) were greater for *P. trichocarpa* compared to *P. taeda*. It is possible that among the shifted communities, those taxa with the ability to interact with two root systems led the greatest change in molecular pathways with *P. trichocarpa* related to *P. taeda* (e.g., mainly pine-associated EMF, Figure 7). Overall, these results show that molecular interactions of these rhizobiomes were stimulated (i.e., more microbial genes were highly expressed) in heterospecific treatments (Figures 5A,D). Consequently, heterospecific species of plant neighbors may lead to a shift in molecular function of the whole fungal rhizobiome in both plants.

### The Effect of Heterospecific Plants on the Gene Expression of Root Fungi

To determine the genes belonging to dominant root fungi that were differentially expressed across the three combination of plant partners, the *FunGene_P. tri* and *FunGene_P. taeda* were assigned to the function (KOG) of closely relative fungal taxa using BlastX (Figure 7). Most of the target genes were assigned to five fungal taxa, including *Rhizophagus*, *Suillus*, *Rhizopogon*, *Cadophora*, and *Ilyonectria*. The relative abundance of the EMF rRNA were directly linked to their functional traits (Figures 3A, 7). For example, the relative abundance of *Suillus* and *Rhizopogon* was enhanced when *P. trichocarpa* was grown with *P. taeda* compared to when *P. trichocarpa* was grown alone (Figure 3A). Consequently, most of genes that were significantly enriched in *P. trichocarpa* in response to *P. taeda* belonged to *Suillus* and *Rhizopogon* (Figure 7B). The molecular mechanisms triggered by certain EMF (i.e., *Suillus*, *Rhizopogon*) were stimulated by means of enrichment of expressed genes, when the plant was grown with heterospecific neighbors compared to when it was grown with conspecifics (Table 1). On the contrary, the expressed genes of other EMF, including *Tuber*, *Hebeloma*, *Cenconccum*, and *Laccaria* were enriched when the plant was grown with conspecific neighbors compared to when it was grown with heterospecifics (Table 1). In addition, many functional genes of fungal taxa that are associated with *Populus* (e.g., *Rhizophagus*, *Mortierella*, and *Ilyonectria*), were also detected when plants were grown with their heterospecific partners (Figure 7A), however, these were not enriched (Figure 7B and Table 1). Taking together, high expression levels of *Pinus*-associated EMF genes were obtained in *Populus* root tissues, which had no visible mycorrhizaes. Despite EMF contribute to over 20% of total fungal rRNA and the total counts of functional genes detected in *Populus* roots regardless neighboring effect (Figures 3A, 7A), less than 2% visible mycorrhizaes were detected in 4-month bioassay system. This indicates that mycorrhizal development was relatively slow in *Populus* root tips, even though the EMF were actively functioning (via gene expression) in and/or on the roots, including *Pinus*-associated EMF. The results imply that certain EMF (e.g., *Suillus* and *Rhizopogon*) may disproportionately contribute to the function of the CMNs. However, changes in gene expression of these *Pinus*-associated EMF on non-host roots may also reflect a response to different biotic and environmental cues or a shift in the relative abundance of this fungal taxa. For example, we cannot exclude the possibilities that the colonization events and the neighboring effects on fungal gene expression can also be mediated directly by the diffusible metabolites between plant species (Farrer and Goldberg, 2011). Compared to most of other EMF, species of *Suillus* and *Rhizopogon* exhibit narrower host ranges across plant family, genus and species levels (Molina and Trappe, 1994; Bruns et al., 2002; Liao et al., 2016). The *Suillus* and *Rhizopogon* detected in this study were from *P. taeda* forest soil and may establish different (non-mycorrhizal) relationships with non-EMF host plants (e.g., *P. trichocarpa*). For example, the hyphae of *Suillus* and *Rhizopogon* may interact with the rhizodeposits released from *P. trichocarpa* to mine nitrogen or/and alter *P. trichocarpa* associated microbiome without colonizing the roots of *P. trichocarpa*. Future studies are needed to understand whether rhizodeposits from *P. trichocarpa* structure the molecular function of *Pinus* EMF specialists.

**TABLE 1:** Hypergeometric test was performed to identify the enrichment of the functional genes of top fungal taxa in associated with neighboring plant traits.

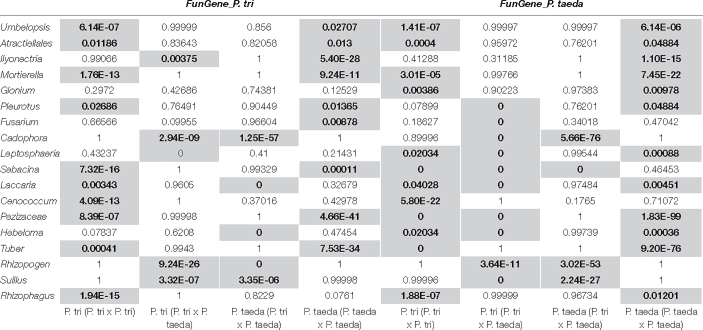

*The number of genes highly expressed in each fungal taxa (Figure 5A) were further compared across different plant combinations using hypergeometric test (Qureshi and Sacan, 2013). The *P*-value in a box <0.05 bold indicates the number of expressed genes (*FunGene_P. tri* and *FunGene_P. taeda*, respectively) for an individual fungal taxa was significantly enriched in a plant combination compared to other combinations.*

In general, similar proportions of genes for biological process (e.g., Zn-finger proteins, transcription initiation factors, ribosomal proteins) were enriched across different groups of fungi when grown with heterospecific plants compared to conspecifics (Supplementary Dataset 4). However, functional annotation of fungal genes indicates that EMF specialists enriched for specific molecular mechanisms in response to heterospecific plant species, only when these fungi were grown with their own host plants. For example, *Suillus* and *Rhizopogon* were both enriched for genes encoding WD-40 repeats (12% of total enriched gene counts) and cytochrome P450 (3% of total enriched gene counts) when interacting with heterospecific hosts compared to conspecifics, but only when these fungal taxa colonized *Pinus* but not *Populus* (Supplementary Dataset 4). Previous studies on *Suillus-Pinus* ectomycorrhizal inoculation and metatranscriptomics showed that genes for WD-40 repeat and cytochrome P450 are involved in host recognition (Liao et al., 2016). Thus, the molecular mechanisms involved in EMF-host recognition/symbiosis may also be altered when a non-host plant is growing nearby.

*Suillus* and *Rhizopogon* also expressed higher proportions of transporter genes (0.6 to 0.9% of significantly expressed genes) during interaction with heterospecific plants compared to conspecifics (Supplementary Datasets 3, 4). For example, the expression of 401 genes of transmembrane proteins assigned to *Suillus* and *Rhizopogon* were significantly increased in *P. trichocarpa* (*P. trichocarpa* × *P. taeda*) compared to *P. trichocarpa* (*P. trichocarpa* × *P. trichocarpa*) roots (Supplementary Dataset 3A1). Of 401 genes, 275 were assigned to the known functions, including the genes that regulate lipid transportation (29 genes), amino acid transportation (24 genes), carbohydrate transport (e.g., monocarboxylate, hexose, sucrose, 19 genes), inorganic ion transport (Ca, Na, P, K, Zn, Cu, 14 genes) and other transporters. This result suggests the potential molecular mechanisms of *Suillus* and *Rhizopogon* involved in fungal nutrient uptake and fungal-*Populus* nutrient exchange while *Pinus* grown nearby. In contrast, different molecular mechanisms were identified for non-EMF in response to heterospecific plants. For example, a high proportion of genes (*FunGene_P. tri*) for transporters (7%), amino acid transporters (4%), transcriptional co-activators (3%) and chitinases (2%) were enriched, when saprotrophs and endophytes interacted with *Pinus* compared to all other plant host combinations (Supplementary Dataset 4). In addition, a higher proportion of genes (*FunGene_P. tri*) encoding for transporters (5%) were expressed when plant pathogens interacted with *Pinus* compared to their conspecific host (*Populus*). Together, these results suggest that activation of transporter genes from multiple fungal taxa with diverse ecological functions is needed to regulate the nutritional flux between organisms. Similarly, changes in the plant community composition may alter the molecular mechanisms of fungal transportation systems and may directly affect source-sink dynamics of any nutritional flux. Overall, these results show that although many differentially expressed genes of fungi for fungal development may be functionally redundant, fungal genes involved in host compatibility and nutrient reallocation are functionally diverse and are differentially regulated depending on plant species.

## Conclusion

Plant growth is influenced by diverse and complex biotic and abiotic drivers. Using metatranscriptomics, we demonstrate that interactions between different plant host species shape their root microbial community structure and molecular function. We observed that a shift in adjacent plant partners had a direct and asymmetric effect on plant growth and rhizosphere community structure (including at the transcriptome level). Key findings in our study include: (a) individual plants can alter the biomass of their heterospecific plant neighbors, presumably through the action of their CMNs, plant-plant competition or the interactive effect of both drivers; (b) *P. trichocarpa* and *P. taeda* growing in a common soil can still establish distinct community structure and functional capacity of their respective root microbiomes; (c) Plant species promotes rhizobiome diversity and the ratio fungal-to-bacterial rRNA reads; (d) Compared to the compatible fungal-plant interaction, fungal specialists (e.g., EMF) can express different gene sets to interact with neighboring incompatible (heterospecific) plant hosts. (e) Heterospecific plant species may enrich the fungal genes for transporter systems across different fungal taxa with diverse ecological functions, compared to conspecifics, which may in turn help to determine changes in nutritional flux between organisms and in soil ecosystems. Together, these results provide an important step to scaling from individual plants to community and ecosystem levels.

## Data Availability Statement

The datasets presented in this study can be found in online repositories. The names of the repository/repositories and accession number(s) can be found below: https://www.ncbi.nlm.nih.gov/, SRP156890.

## Author Contributions

H-LL, GB, KH, JL, CS, GT, FM, and RV: design and perform the experiments. H-LL, SW, and K-HC: data assembly and analysis. AK, KB, and IG: genome references publication (Joint Genome Institute). H-LL, GB, and RV: manuscript writing. All authors contributed to manuscript editing.

## Conflict of Interest

The authors declare that the research was conducted in the absence of any commercial or financial relationships that could be construed as a potential conflict of interest.

## Publisher’s Note

All claims expressed in this article are solely those of the authors and do not necessarily represent those of their affiliated organizations, or those of the publisher, the editors and the reviewers. Any product that may be evaluated in this article, or claim that may be made by its manufacturer, is not guaranteed or endorsed by the publisher.

## References

[B1] Anders S. Huber W. (2010). Differential expression analysis for sequence count data. *Nat. Prec.* 2010:4282. 10.1038/npre.2010.4282.1 PMC3218662 20979621

[B2] Andronov E. E. Igolkina A. A. Kimeklis A. K. Vorobyov N. I. Provorov N. A. (2015). Characteristics of natural selection in populations of nodule bacteria (Rhizobium leguminosarum) interacting with different host plants. *Russ. J. Genet.* 51 949–956. 10.1134/S1022795415100026 27169225

[B3] Aučina A. Rudawska M. Leski T. Ryliškis D. Pietras M. Riepšas E. (2011). Ectomycorrhizal fungal communities on seedlings and conspecific trees of Pinus mugo grown on the coastal dunes of the Curonian Spit in Lithuania. *Mycorrhiza* 21 237–245. 10.1007/s00572-010-0341-3 20938693 PMC3058383

[B4] Bais H. P. Weir T. L. Perry L. G. Gilroy S. Vivanco J. M. (2006). The role of root exudates in rhizosphere interactions with plants and other organisms. *Annu. Rev. Plant Biol.* 57 233–266. 10.1146/annurev.arplant.57.032905.105159 16669762

[B5] Bauer D. F. (1972). Constructing Confidence Sets Using Rank Statistics. *J. Am. Stat. Assoc.* 67 687–690. 10.1080/01621459.1972.10481279

[B6] Baum C. Stetter U. Makeschin F. (2002). Growth response of Populus trichocarpa to inoculation by the ectomycorrhizal fungus Laccaria laccata in a pot and a field experiment. *Forest Ecol. Manag.* 163 1–8. 10.1016/S0378-1127(01)00534-5

[B7] Bjorkman E. R. I. K. (1960). Monotropa hypopitys L.-an epiparasite on tree roots. *Physiol. Plant.* 13 308–327. 10.1111/j.1399-3054.1960.tb08034.x

[B8] Bonito G. Reynolds H. Robeson M. S. II Nelson J. Hodkinson B. P. Tuskan G. (2014). Plant host and soil origin influence fungal and bacterial assemblages in the roots of woody plants. *Mol. Ecol.* 23 3356–3370. 10.1111/mec.12821 24894495

[B9] Bruns T. D. Bidartondo M. I. Taylor D. L. (2002). Host specificity in ectomycorrhizal communities: what do the exceptions tell us? *Integr. Comp. Biol.* 42 352–359. 10.1093/icb/42.2.352 21708728

[B10] Bücking H. Mensah J. A. Fellbaum C. R. (2016). Common mycorrhizal networks and their effect on the bargaining power of the fungal partner in the arbuscular mycorrhizal symbiosis. *Commun. Integr. Biol.* 9:e1107684. 10.1080/19420889.2015.1107684 27066184 PMC4802747

[B11] Cole J. R. Wang Q. Fish J. A. Chai B. McGarrell D. M. Sun Y. (2014). Ribosomal Database Project: data and tools for high throughput rRNA analysis. *Nucleic Acids Res.* 42 D633–D642. 10.1093/nar/gkt1244 24288368 PMC3965039

[B12] Compant S. Samad A. Faist H. Sessitsch A. (2019). A review on the plant microbiome: Ecology, functions, and emerging trends in microbial application. *J. Advert. Res.* 19 29–37. 10.1016/j.jare.2019.03.004 31341667 PMC6630030

[B13] Craine J. M. Dybzinski R. (2013). Mechanisms of plant competition for nutrients, water and light. *Funct. Ecol.* 27 833–840. 10.1111/1365-2435.12081

[B14] Danielsen L. Lohaus G. Sirrenberg A. Karlovsky P. Bastien C. Pilate G. (2013). Ectomycorrhizal colonization and diversity in relation to tree biomass and nutrition in a plantation of transgenic poplars with modified lignin biosynthesis. *PLoS One* 8:e59207. 10.1371/journal.pone.0059207 23516610 PMC3596300

[B15] Deshpande V. Wang Q. Greenfield P. Charleston M. Porras-Alfaro A. Kuske C. R. (2016). Fungal identification using a Bayesian classifier and the Warcup training set of internal transcribed spacer sequences. *Mycologia* 108 1–5. 10.3852/14-293 26553774

[B16] Dickie I. A. Koide R. T. Steiner K. C. (2002). Influences of established trees on mycorrhizas, nutrition, and growth of Quercus rubra seedlings. *Ecol. Monogr.* 72 505–521. 10.1890/0012-9615(2002)072[0505:IOETOM]2.0.CO;2

[B17] Dighton J. (2009). *Mycorrhizae in Encyclopedia of microbiology.* Amsterdam: Elsevier Inc, 153–162. 10.1016/B978-012373944-5.00327-8

[B18] Dijkstra F. A. Carrillo Y. Pendall E. Morgan J. A. (2013). Rhizosphere priming: a nutrient perspective. *Front. Microbiol.* 4:216. 10.3389/fmicb.2013.00216 23908649 PMC3725428

[B19] el Zahar Haichar F. Marol C. Berge O. Ignacio Rangel-Castro J. Prosser J. I Balesdent J. (2008). Plant host habitat and root exudates shape soil bacterial community structure. *ISME J.* 2 1221–1230. 10.1038/ismej.2008.80 18754043

[B20] Farrer E. C. Goldberg D. E. (2011). Patterns and mechanisms of conspecific and heterospecific interactions in a dry perennial grassland. *J. Ecol.* 99 265–276. 10.1111/j.1365-2745.2010.01734.x

[B21] Fellbaum C. R. Mensah J. A. Cloos A. J. Strahan G. E. Pfeffer P. E. Kiers E. T. (2014). Fungal nutrient allocation in common mycorrhizal networks is regulated by the carbon source strength of individual host plants. *New Phytol.* 203 646–656. 10.1111/nph.12827 24787049

[B22] Felten J. Kohler A. Morin E. Bhalerao R. P. Palme K. Martin F. (2009). The ectomycorrhizal fungus Laccaria bicolor stimulates lateral root formation in poplar and Arabidopsis through auxin transport and signaling. *Plant Physiol.* 151 1991–2005. 10.1104/pp.109.147231 19854859 PMC2785963

[B23] Futai K. Taniguchi T. Kataoka R. (2008). “Ectomycorrhizae and Their Importance in Forest Ecosystems,” in *Mycorrhizae: Sustainable Agriculture and Forestry*, eds Siddiqui Z. A. Akhtar M. S. Futai K. (Dordrecht: Springer), 241–285.

[B24] Garbeva P. van Elsas J. D. van Veen J. A. (2008). Rhizosphere microbial community and its response to plant species and soil history. *Plant Soil* 302 19–32. 10.1007/s11104-007-9432-0

[B25] Gorzelak M. A. Asay A. K. Pickles B. J. Simard S. W. (2015). Inter-plant communication through mycorrhizal networks mediates complex adaptive behaviour in plant communities. *AoB Plants* 7:50. 10.1093/aobpla/plv050 25979966 PMC4497361

[B26] Grayston S. J. Vaughan D. Jones D. (1997). Rhizosphere carbon flow in trees, in comparison with annual plants: the importance of root exudation and its impact on microbial activity and nutrient availability. *Appl. Soil Ecol.* 5 29–56. 10.1016/S0929-1393(96)00126-6

[B27] Helgason T. Merryweather J. W. Denison J. Wilson P. Young J. P. W. Fitter A. H. (2002). Selectivity and functional diversity in arbuscular mycorrhizas of co-occurring fungi and plants from a temperate deciduous woodland. *J. Ecol.* 90 371–384. 10.1046/j.1365-2745.2001.00674.x

[B28] Helgason T. Merryweather J. W. Young J. P. W. Fitter A. H. (2007). Specificity and resilience in the arbuscular mycorrhizal fungi of a natural woodland community. *J. Ecol.* 95 623–630. 10.1111/j.1365-2745.2007.01239.x

[B29] Jones P. Garcia B. J. Furches A. Tuskan G. A. Jacobson D. (2019). Plant Host-Associated Mechanisms for Microbial Selection. *Front. Plant Sci.* 10:862. 10.3389/fpls.2019.00862 31333701 PMC6618679

[B30] Kadowaki K. Yamamoto S. Sato H. Tanabe A. S. Hidaka A. Toju H. (2018). Mycorrhizal fungi mediate the direction and strength of plant–soil feedbacks differently between arbuscular mycorrhizal and ectomycorrhizal communities. *Comm. Biol.* 1 1–11. 10.1038/s42003-018-0201-9 PMC6244237 30480098

[B31] Khare E. Mishra J. Arora N. K. (2018). Multifaceted Interactions Between Endophytes and Plant: Developments and Prospects. *Front. Microb.* 9:2732. 10.3389/fmicb.2018.02732 30498482 PMC6249440

[B32] Khasa P. D. Chakravarty P. Robertson A. Thomas B. R. Dancik B. P. (2002). The mycorrhizal status of selected poplar clones introduced in Alberta. *Biomass Bioener.* 22 99–104. 10.1016/S0961-9534(01)00072-1

[B33] Klein T. Siegwolf R. T. W. Körner C. (2016). Belowground carbon trade among tall trees in a temperate forest. *Science* 352 342–344. 10.1126/science.aad6188 27081070

[B34] Li H. Handsaker B. Wysoker A. Fennell T. Ruan J. Homer N. (2009). The Sequence Alignment/Map format and SAMtools. *Bioinformatics* 25 2078–2079. 10.1093/bioinformatics/btp352 19505943 PMC2723002

[B35] Liao H.-L. Bonito G. Alejandro Rojas J. Hameed K. Wu S. Schadt C. W. (2019). Fungal Endophytes of Populus trichocarpa Alter Host Phenotype, Gene Expression, and Rhizobiome Composition. *Mole. Plant-Microbe Interact.* 32 853–864. 10.1094/MPMI-05-18-0133-R 30699306

[B36] Liao H.-L. Chen Y. Bruns T. D. Peay K. G. Taylor J. W. Branco S. (2014). Metatranscriptomic analysis of ectomycorrhizal roots reveals genes associated with Piloderma-Pinus symbiosis: improved methodologies for assessing gene expression in situ. *Environ. Microbiol.* 16 3730–3742. 10.1111/1462-2920.12619 25186788

[B37] Liao H.-L. Chen Y. Vilgalys R. (2016). Metatranscriptomic Study of Common and Host-Specific Patterns of Gene Expression between Pines and Their Symbiotic Ectomycorrhizal Fungi in the Genus Suillus. *PLoS Genet.* 12:e1006348. 10.1371/journal.pgen.1006348 27736883 PMC5065116

[B38] Liu Y. Li X. Kou Y. (2020). Ectomycorrhizal Fungi: Participation in Nutrient Turnover and Community Assembly Pattern in Forest Ecosystems. *For. Trees Livelihoods* 11:453.

[B39] Malik A. A. Chowdhury S. Schlager V. Oliver A. Puissant J. Vazquez P. G. M. (2016). Soil Fungal:Bacterial Ratios Are Linked to Altered Carbon Cycling. *Front. Microb.* 7:1247. 10.3389/fmicb.2016.01247 27555839 PMC4977315

[B40] Marupakula S. Mahmood S. Finlay R. D. (2016). Analysis of single root tip microbiomes suggests that distinctive bacterial communities are selected by P inus sylvestris roots colonized by different ectomycorrhizal fungi. *Environ. Microbiol.* 18 1470–1483. 10.1111/1462-2920.13102 26521936

[B41] Molina R. (1992). *Specificity Phenomena in Mycorrhizal Symbioses: Community-Ecological Consequences and Practical Implications Randy Molina, Hugues Massicotte, and James M. Trappe. Mycorrhizal functioning: an integrative plant-fungal process.* New York, NY: Springer, 357.

[B42] Molina R. (1994). The role of mycorrhizal symbioses in the health of giant redwoods and other forest ecosystems1. *Proc.* S*ymp. Giant Seq.* 1994 78–81.

[B43] Molina R. Trappe J. M. (1994). Biology of the ectomycorrhizal genus, Rhizopogon. I. Host associations, host-specificity and pure culture syntheses. *New Phytol.* 126 653–675. 10.1111/j.1469-8137.1994.tb02961.x

[B44] Morris M. H. Smith M. E. Rizzo D. M. Rejmánek M. Bledsoe C. S. (2008). Contrasting ectomycorrhizal fungal communities on the roots of co-occurring oaks (Quercus spp.) in a California woodland. *New Phytol.* 178 167–176. 10.1111/j.1469-8137.2007.02348.x 18194145

[B45] Nara K. (2006). Ectomycorrhizal networks and seedling establishment during early primary succession. *New Phytol.* 169 169–178. 10.1111/j.1469-8137.2005.01545.x 16390428

[B46] Nguyen N. H. Bruns T. D. (2015). The Microbiome of Pinus muricata Ectomycorrhizae: Community Assemblages, Fungal Species Effects, and Burkholderia as Important Bacteria in Multipartnered Symbioses. *Microb. Ecol.* 69 914–921. 10.1007/s00248-015-0574-y 25687126

[B47] Nguyen N. H. Song Z. Bates S. T. Branco S. Tedersoo L. Menke J. (2016). FUNGuild: An open annotation tool for parsing fungal community datasets by ecological guild. *Fungal Ecol.* 20 241–248. 10.1016/j.funeco.2015.06.006

[B48] Ortíz-Castro R. Contreras-Cornejo H. A. Macías-Rodríguez L. López-Bucio J. (2009). The role of microbial signals in plant growth and development. *Plant Signal. Behav.* 4 701–712. 10.4161/psb.4.8.9047 19820333 PMC2801380

[B49] Pausch J. Zhu B. Kuzyakov Y. Cheng W. (2013). Plant inter-species effects on rhizosphere priming of soil organic matter decomposition. *Soil Biol. Biochem.* 57 91–99. 10.1016/j.soilbio.2012.08.029

[B50] Plett J. M. Kemppainen M. Kale S. D. Kohler A. Legué V. Brun A. (2011). A secreted effector protein of Laccaria bicolor is required for symbiosis development. *Curr. Biol.* 21 1197–1203. 10.1016/j.cub.2011.05.033 21757352

[B51] Policelli N. Bruns T. D. Vilgalys R. Nuñez M. A. (2019). Suilloid fungi as global drivers of pine invasions. *New Phytol.* 222 714–725. 10.1111/nph.15660 30586169

[B52] Qureshi R. Sacan A. (2013). Weighted set enrichment of gene expression data. *BMC Syst. Biol.* 7 (Suppl. 4):S10. 10.1186/1752-0509-7-S4-S10 24565001 PMC3854649

[B53] Robinson C. H. Smith S. E. Read D. J. (1997). Mycorrhizal Symbiosis, 2nd edn. *J. Ecol.* 85:925. 10.2307/2960617

[B54] Selosse M.-A. Richard F. He X. Simard S. W. (2006). Mycorrhizal networks: des liaisons dangereuses? *Trends Ecol. Evol.* 21 621–628. 10.1016/j.tree.2006.07.003 16843567

[B55] Shakya M. Gottel N. Castro H. Yang Z. K. Gunter L. Labbé J. (2013). A multifactor analysis of fungal and bacterial community structure in the root microbiome of mature Populus deltoides trees. *PLoS One* 8:e76382. 10.1371/journal.pone.0076382 24146861 PMC3797799

[B56] Simard S. W. Beiler K. J. Bingham M. A. Deslippe J. R. Philip L. J. Teste F. P. (2012). Mycorrhizal networks: Mechanisms, ecology and modelling. *Fungal Biol. Rev.* 26 39–60. 10.1016/j.fbr.2012.01.001

[B57] Simard S. W. Perry D. A. Jones M. D. Myrold D. D. Durall D. M. Molina R. (1997). Net transfer of carbon between ectomycorrhizal tree species in the field. *Nature* 388 579–582. 10.1038/41557

[B58] Smith S. E. Read D. J. (1997). *Mycorrhizal Symbiosis.* 2nd Edn. London: Academic Press.

[B59] Smith S. E. Read D. (2008). Structure and development of ectomycorrhizal roots. *Mycorrhizal Symb.* 2008:191. 10.1016/B978-012370526-6.50008-8

[B60] Song Y. Y. Simard S. W. Carroll A. Mohn W. W. Zeng R. S. (2015). Defoliation of interior Douglas-fir elicits carbon transfer and stress signalling to ponderosa pine neighbors through ectomycorrhizal networks. *Sci. Rep.* 5:8495. 10.1038/srep08495 25683155 PMC4329569

[B61] Steinauer K. Chatzinotas A. Eisenhauer N. (2016). Root exudate cocktails: the link between plant diversity and soil microorganisms? *Ecol. Evol.* 6 7387–7396. 10.1002/ece3.2454 28725406 PMC5513276

[B62] Tedersoo L. Jairus T. Horton B. M. Abarenkov K. Suvi T. Saar I. (2008). Strong host preference of ectomycorrhizal fungi in a Tasmanian wet sclerophyll forest as revealed by DNA barcoding and taxon-specific primers. *New Phytol.* 180 479–490. 10.1111/j.1469-8137.2008.02561.x 18631297

[B63] Teste F. P. Simard S. W. (2008). Mycorrhizal networks and distance from mature trees alter patterns of competition and facilitation in dry Douglas-fir forests. *Oecologia* 158 193–203. 10.1007/s00442-008-1136-5 18781333

[B64] Treseder K. K. Cross A. (2006). Global Distributions of Arbuscular Mycorrhizal Fungi. *Ecosystems* 9 305–316. 10.1007/s10021-005-0110-x

[B65] van der Heijden M. G. A. Horton T. R. (2009). Socialism in soil? The importance of mycorrhizal fungal networks for facilitation in natural ecosystems. *J. Ecol.* 97 1139–1150. 10.1111/j.1365-2745.2009.01570.x

[B66] Verbon E. H. Liberman L. M. (2016). Beneficial Microbes Affect Endogenous Mechanisms Controlling Root Development. *Trends Plant Sci.* 21 218–229. 10.1016/j.tplants.2016.01.013 26875056 PMC4772406

[B67] Viebahn M. Veenman C. Wernars K. van Loon L. C. Smit E. Bakker P. A. H. M. (2005). Assessment of differences in ascomycete communities in the rhizosphere of field-grown wheat and potato. *FEMS Microbiol. Ecol.* 53 245–253. 10.1016/j.femsec.2004.12.014 16329944

[B68] Vilgalys R. Hester M. (1990). Rapid genetic identification and mapping of enzymatically amplified ribosomal DNA from several Cryptococcus species. *J. Bacteriol.* 172 4238–4246. 10.1128/jb.172.8.4238-4246.1990 2376561 PMC213247

[B69] Vincenot L. Selosse M.-A. (2017). “Population Biology and Ecology of Ectomycorrhizal Fungi,” in *Biogeography of Mycorrhizal Symbiosis*, ed. Tedersoo L. (Cham: Springer International Publishing), 39–59. 10.1007/978-3-319-56363-3_2

[B70] Wang Q. Liu J. Zhu H. (2018). Genetic and Molecular Mechanisms Underlying Symbiotic Specificity in Legume-Rhizobium Interactions. *Front. Plant Sci.* 9:313. 10.3389/fpls.2018.00313 29593768 PMC5854654

[B71] Weremijewicz J. Sternberg L. D. S. L. O. R. Janos D. P. (2016). Common mycorrhizal networks amplify competition by preferential mineral nutrient allocation to large host plants. *New Phytol.* 212 461–471. 10.1111/nph.14041 27265515

[B72] Yin L. Dijkstra F. A. Wang P. Zhu B. Cheng W. (2018). Rhizosphere priming effects on soil carbon and nitrogen dynamics among tree species with and without intraspecific competition. *New Phytol.* 218 1036–1048. 10.1111/nph.15074 29512165

