# Supplementary material for: Heterospecific Neighbor Plants Impact Root Microbiome Diversity and Molecular Function of Root Fungi
Source: Front Microbiol. 2021 Nov 4;12:680267. doi: 10.3389/fmicb.2021.680267 (PMC8601753; doi:10.3389/fmicb.2021.680267)
Supplement: Supplementary file 1 [file Data_Sheet_1.pdf]

Supporting Information (SI)

Table of contents

A. SI Text

A1. Plant materials and growing conditions prior to soil bioassay

A2. RNAseq data assembly and analysis

A2_1: Analysis of fungal rRNA D1D2 and bacterial 16S reads

A2_2: Analysis of plant associated genes

A2_3: Analysis of fungal associated genes

A2_4: Fungal gene analysis and annotation

A3. DNA/cDNA amplicon sequence analysis of fungal rRNA

B. Supplementary Figures: S1 - S6

C. Supplementary Tables: S1- S3

A1. *Plant materials and growing conditions prior to soil bioassay*

Pinus taeda seeds were purchased from Sheffield's Seed Co., Inc. (Locke, NY, seed ID 090152, collected from Georgia, USA). Seeds were surface sterilized in 10% bleach for 10 min, suspended in sterilized water overnight, and stratified at 4 °C for 30 days prior to germination. Germinated seedlings were planted in sterilized sand and watered with sterile water. *Populus trichocarpa* genotype BESC4 was used for this study. Twelve-inch long vegetative cuttings of *P. trichocarpa* were submerged in sterile distilled water for three days, with daily water changes. Cuttings were planted into sterile sand to root under fluorescent lighting with regular watering. Abiotic factors that regulate growth conditions were controlled in the growth chamber, including

water, temperature, humidity, and lighting. Plants were grown in a growth chamber at 25 °C, 80% humidity, and fluorescent light at 200 µmol m-2s-1 for 12 hours per day. After two months of growth, *P. taeda* and *P. trichocarpa* plants were used for bioassay experiments.

A2. ***RNAseq data assembly and analysis***

Illumina RNAseq (Hiseq 2000) generated 40 million (M) reads for individual samples (Dataset S1). On average, over 92% of the reads passed quality control of the FastQ filter pipeline(Blankenberg *et al.*, 2010). Raw reads were filtered based upon the quality scores > = 20. Reads containing ambiguous (N) were removed (Fig. S2, Step S1). Fig. S2 described the summarized workflow for RNAseq data assembly. Tools used for this workflow [nine steps (S1-S9)] are listed below and Fig. S2.

A2_1: PERMANOVA Analysis of fungal rRNA D1D2 and bacterial 16S reads (Step S2-S5 in Fig. S2; Fig. 3; Fig. 4; Table S3; Dataset S2)

The poly-A selected cDNA library approach was used to identify fungal communities present in each sample (Illumina Inc.). Even though the RNAseq library consisted mostly of poly-A selected cDNA reads, a portion of fungal ribosomal RNA sequences were present after root cDNA library preparation. The number of D1/D2 reads versus the total reads of individual samples (relative value) was used to generate Fig. 3a, Fig. 4a and Fig. 4b.
We generated a custom LSU D1/D2 reference for fungal taxa identification. Briefly, fungal ribosomal large subunit DNA sequences were downloaded from RDP classifier (Cole *et al.*,

2014; Deshpande *et al.*, 2016), NCBI, and the sequences of fungal endophytes that were isolated from the *Populus* mycobiome (Bonito *et al.*, 2016). The LSU D1/D2 sequence database was then created corresponding to the D1/D2 region. Here, sequences comprising the D1/D2 region were extracted by being matched (>=80% match) to the flanking region, LR3 (5'-CCGTGTTTCAAGACGGG-3') and LROR (5'- ACCCGCTGAACTTAAGC-3') (Vilgalys & Hester, 1990). Reference sequences that contained ambiguous (N) were removed. A total of 29,342 representative D1/D2 sequences were used as the reference database for fungal identification.

To sort out reads belonging to bacterial 16S rRNA and fungal LSU D1/D2 reads, filtered reads (~31 million reads per sample) were mapped onto reference sequences with Bowtie using default settings (Langmead & Salzberg, 2012) (Fig. S2, Step 3). Reads belonging to fungal LSU D1/D2 or bacterial 16S were obtained via reference-based mapping (Bowtie2, SAMtools), reports of BAM index (SAMtools idxstats) and bam-to-fastq format conversion (Picard) using default settings (Li *et al.*, 2009). The alignments selected in Bowtie 2 for paired-end include -I/--minins <int> as 0; -X/-maxins <int> as 500; --fr; --dovetail mates cannot dovetail in a concordant alignment; --no-contain a mate can contain the other in a concordant alignment; --no-overlap mates can overlap in a concordant alignment. The options of Picard used to format SAM include FilterSamReads, and SamToFastq. Short reads were assigned to taxon using RDP/fungal LSU (for rRNA LSU D1/D2 read) and RDP bacterial 16S rDNA (for 16S read) (Cole *et al.*, 2014) with cutoff >=0.6 (Fig S2, Step 4). An average of 53,943 and 144,014 fungal LSU D1/D2 short read region (~200bp) and prokaryotic 16S rRNA reads were sorted out and used to predict the transcript abundance of the dominant root prokaryotes (Fig. S2; Dataset S1 and S2). The distribution of fungal LSU D1/D2 and bacterial 16S rRNA reads across the treatments ranging

from 8,000 to 162,000 reads (for D1/D2) and 5,000 to 555,000 (16S) respectively (Dataset S1). An additional step of BLASTing extracted reads to *P. trichocarpa* and *P. taeda* databases was performed to filter out reads belonging to plastid and mitochondrial rRNA genes. To generate Fig. 3a and 3b, the percentage of reads was calculated according to the ratio of reads from individual taxa vs. total rRNA reads obtained from the individual samples (Fig. S2, Step 4). The ecological function of each fungal group was assigned according to FUNGuild (Nguyen et al., 2016). Differences in community composition among the comparisons were tested using permutational multivariate analysis of variance (PERMANOVA). Results for PERMANOVA were corrected for multiple comparison using false discovery rate (FDR). P-value were calculated based on pseudo-F statistics, and results with $P \leq 0.05$ were considered as statistically significant (Results shown in Fig. 4a to 4d; Table S3; Dataset S2B and S2D). Both non-metric multidimensional scaling (NMDS) and PERMANOVA were performed using vegan package version 2.5.3 in R (3.5.1). They are performed using metaMDS and adonis functions respectively. The Zero-adjusted for the paired-comparisons (Dataset S2B and S2D) was applied according to Clarke et al (Clarke *et al.*, 2006).

A2_2: Analysis of plant-associated genes (Step S6 in Fig. S2)

Remaining unmapped reads (approximately 30-million) were mapped onto reference sequences of *P. trichocarpa* and *P. taeda*. TopHat package (Trapnell *et al.*, 2009) was used for *P. trichocarpa* genome mapping and Bowtie2 package was used for *P. taeda* transcriptome mapping (Fig S2, Step 6). Default settings were applied for both packages except a minimal intron setting of “20” was used for *P. trichocarpa* root samples. The reference used in this study

include *P. trichocarpa* 210 v3(Tuskan *et al.*, 2006) and *P. taeda* EST database (NCBI, 328,662 contigs). Expression patterns of plant hosts in response to neighbor effects will be analyzed in future work.

A2_3: Analysis of fungal associated genes (Step S7-S9 in Fig. S2)

We expected different plant species and plant partner combinations to harbor distinct microbial communities and to shape the function of those microbes. We applied our bioinformatic workflow (Fig, S1 Step 5; Fig. S2, Step S7-S9) to extract reads belonging to functional genes of *P. trichocarpa*-associated fungi (*FunGene_P.tri*) & *P. taeda*-associated fungi (*FunGene_P.taeda*) respectively. In Fig. S2 Step 7, unmapped reads (from Fig. S2, Step 6) for individual *P. trichocarpa* root samples were pooled and a sub-workflow (Fig. S3A) was applied to generate *de novo*-based references for *P. trichocarpa* associated fungal transcripts (481,969 contigs). Another sub-workflow was applied for *P. taeda* root samples to generate *de novo*-based references for *P. taeda* associated fungal transcripts (155,286 contigs) (Fig. S3b). More contigs for *P. trichocarpa* associated fungi were expected since a higher diversity of dominant fungal taxa was found on *P. trichocarpa* roots compared *P. taeda* root tips. Unmapped reads of individual root samples (after Fig. S2, Step 6) that were expected to belong to root fungi were mapped to these *de novo*-based references (Fig. S2, Step 9) with Bowtie2. DESeq packages were applied for read normalization. Comparative transcriptomics was applied to identify relative expression of *P. trichocarpa* and *P. taeda* fungal genes in response to different plant species and plant partners (Fig 5 & 6) (FDR<0.05, > 2-fold, Wilcoxon signed-rank test and Benjamini-

Hochberg). Plots (Figure 5 & 6) and heatmaps (Fig. 7) were generated with statistical packages in R (R Development Core Team, 2003).

A2_4: Challenge of fungal gene analysis and annotation

The limitation of genome databases makes it challenge to characterize the function of dominant fungal taxa. For example, pine roots collected for this study were colonized by EMF (Table S1) and were shown to be dominated by *Rhizopogen* and *Suillus* (Fig. 3). Our previous studies showed over 30% RNAseq reads belonging to EMF should be recovered from EMF root tips with fungal genome databases available (Liao *et al.*, 2016). However, in this study, much fewer EMF reads (~1%) from pine roots were recovered from genome-based analysis (Fig. S6). It indicates a limitation of current genome databases, which must be lacking taxa closely related to the dominant fungal species that colonize pine roots. To study the compatibility of *Pinus*-associated (EMF) function on their host (*P. taeda*) and non-host (*P. trichocarpa*), *de novo* assembly was applied to recover fungal genes (Fig. S2). With this strategy over 30% of the total reads, representing 481,969 and 155,286 fungal gene contigs, were detected from *Populus* and pine roots, respectively (Fig. S3). We used these gene contigs as databases to identify key *Pinus*-associated EMF genes responding to host and non-host factors (Fig. 5). To study the transcriptomic function of dominant phytobiomes involved in fungal-plant communication, 24 fungal genomes were used as references to BLAST to RNAseq reads. Genomes of taxa closely related to the highly active fungal taxa in root and soil samples were selected as references. In general, genome-based mapping recovered abundant functional reads (for most of the samples >0.5% of total reads) that belonged to *Rhizopogon*, *Hebeloma*, *Tuber*, *Cenococcum*,

Rhizophagus, *Leptosphaeria*, *Mortierella*, *Pleurotus*, *Glonium*, and *Atractiella* (Fig. S6). To generate an amino acid database for BlastX, amino acid sequences from 24 fungal genomes were pooled, including Suihi1, Suidec1, Suigr1, Suibr2(Branco *et al.*, 2015), Suilu1(Kohler *et al.*, 2015), Rhivi1(Mujic *et al.*, 2017), Tubme1(Martin *et al.*, 2010), Suiame1, Suipic1, Hebcy2(Kohler *et al.*, 2015), Gloin1(Tisserant *et al.*, 2013), Lepmu1(Rouxel *et al.*, 2011), Morel1(Uehling *et al.*, 2017), Umbra1, PleosPC15(Riley *et al.*, 2014; Alfaro *et al.*, 2016; Castanera *et al.*, 2016), Glost2(Cole *et al.*, 2014), Cenge3(Peter *et al.*, 2016), Atrsp2, llysp1, Cadsp1(Knapp *et al.*, 2018), Sebve1(Kohler *et al.*, 2015), Fusgr1(Cuomo *et al.*, 2007), Lacam2(Kohler *et al.*, 2015), Lacbi2(Martin *et al.*, 2008) = 24F_aa.fasta. The use of genome reference sequences from fungal taxa presented on *Populus* sites aided the identification of gene expression in *Populus*-associated microbiomes that might have been missed in Fig. 3 due to the lack of their rRNA database in RDP classifier (Wang *et al.*, 2007). Genes that were significantly expressed in associating with neighbor effects (color dots in Fig. 5) were assigned to function (% ID >50, e<10^-3, alignment length>30) (Fig. S3; SI text A2_4; Dataset S3).

A3. cDNA amplicon sequence analysis of fungal rRNA

The same RNA extractions for RNAseq (Fig. S2, Step3) were used for cDNA-amplicon sequencing. cDNAs were prepared with the SuperScript II Reverse Transcriptase kit (Sigma) and LR3 primer following the manufacturer's instruction. A 3-step PCR protocol with LR0R and LR3 primer pair and barcodes was performed (Johansen *et al.*, 2016). PCR products were multiplexed and sequenced on the Illumina MiSeq platform (v2, 250PE) at Duke Genomic

Center of Biology, USA. Samples with inadequate RNAs left were excluded. Reads obtained were first processed with Cutadapt (Martin, 2011) to remove adaptors. Reads were then quality checked and trimmed to 140 bp in USEARCH 8 with an error rate < 0.75(Edgar, 2013). The number of reads kept for downstream analysis ranged between 20,847 to 188,703. In average, the qualified reads recovered from cDNA amplicon sequencing recovered across the treatments include 161,528 for *Populus* growing near *Pinus*, 70,887 for *Pinus* growing near *Populus*, and 132,806 for *Pinus* growing near *Pinus*. Singletons were removed prior to sequence clustering at 97% similarity in USEARCH 8. Short reads were mapped to representative OTU sequences. OTU tables were prepared in QIIME(Caporaso *et al.*, 2010). Taxonomy assignments were performed with the RDP classifier and an 80% bootstrap value cut-off.

B. Supplementary Figures: S1 to S6

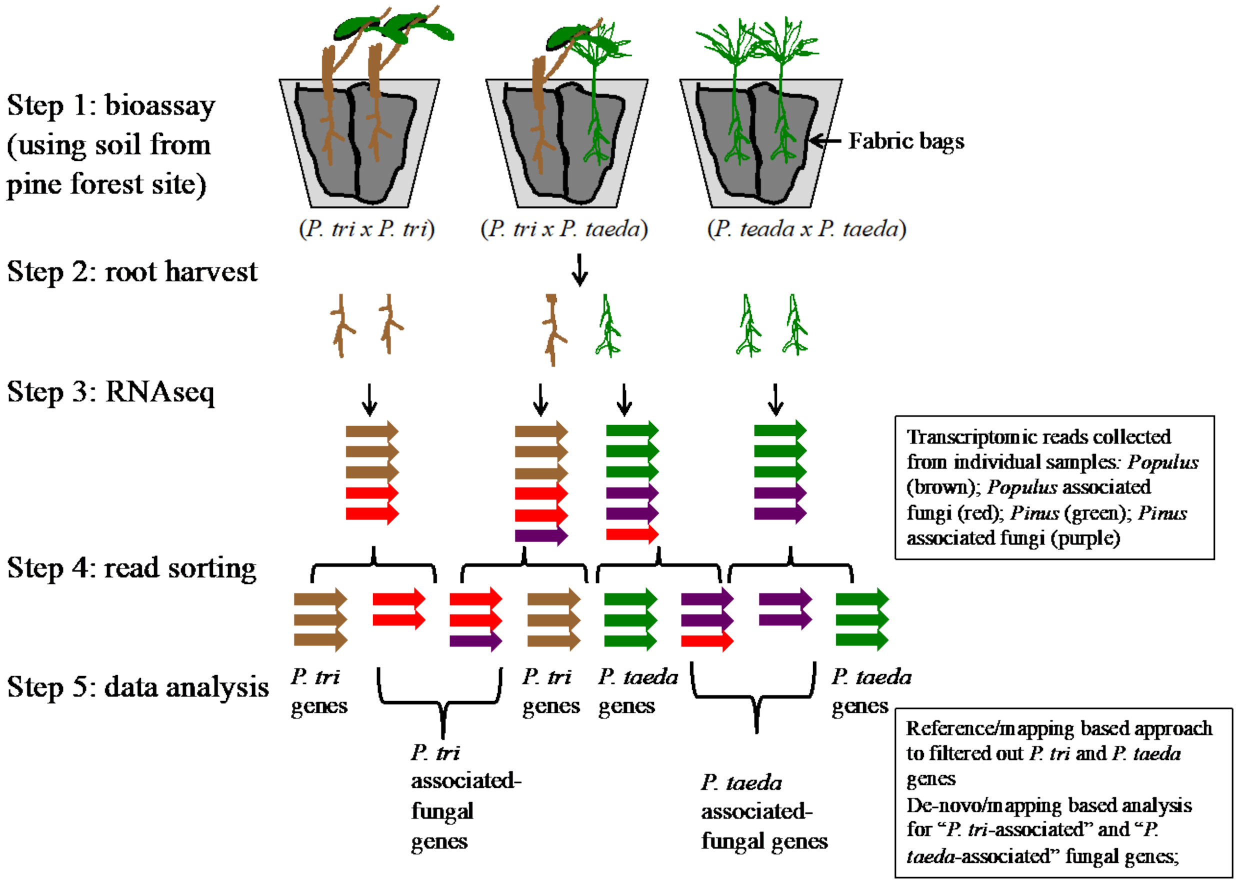

Fig S1. An illustration summarizing the experimental design for plant bioassay experiments.

Briefly, soil collected from *Pinus taeda* forest sites (Durham, NC, USA) was used to grow *P. taeda* (native-hosts) and *Populus trichocarpa* (*P. tri*, non-native hosts) in three combinations, including *Populus* growing with *Populus*, *Populus* growing with *Pinus*, and *Pinus* growing with *Pinus* (Step 1). Root samples were harvested 4-months after planting (Step 2). RNAseq was performed to obtain reads from these root samples (Step 3). To study the community structure and transcriptional abundance of root microbiomes, DNA and RNA from the same root samples was purified (following the method published by Liao et al.(Liao *et al.*, 2014). We applied Illumina RNA HiSeq for deep sequencing of polyA-enriched RNA from individual root samples. Reads of bacterial 16S rRNA and fungal rRNA LSU D1D2 regions that leaked through polyA-

selection enabled us to identify the transcriptomic abundance of microbes in the root tissue. The computational pipeline applied for this step is described in SI text A2_1. To identify key genes involved in plant-root fungal microbiome interactions, transcript reads of RNAseq were sorted based upon *de novo* (for fungal genes) and reference (for plant genes) approaches (Step 4 & 5; SI text A2_2 & A2_3). Through these approaches, we were able to compare and identify genes in root samples belonging to *P. trichocarpa*-associated fungi (*FunGene_P.tri*) and *P. taeda-associated* fungi (*FunGene_P.taeda*). Comparative transcriptomics was used to identify key fungal genes involved in plant-fungal interactions. Here, we focused solely on gene expression patterns underlying the fungal partner. Plant response mechanisms to neighbor effects will be addressed in future studies. We applied Illumina Miseq amplicon sequence strategies to target LSU/D1D2 region of cDNA to identify and compare key fungal taxa from the individual root samples (detailed method in SI text A3).

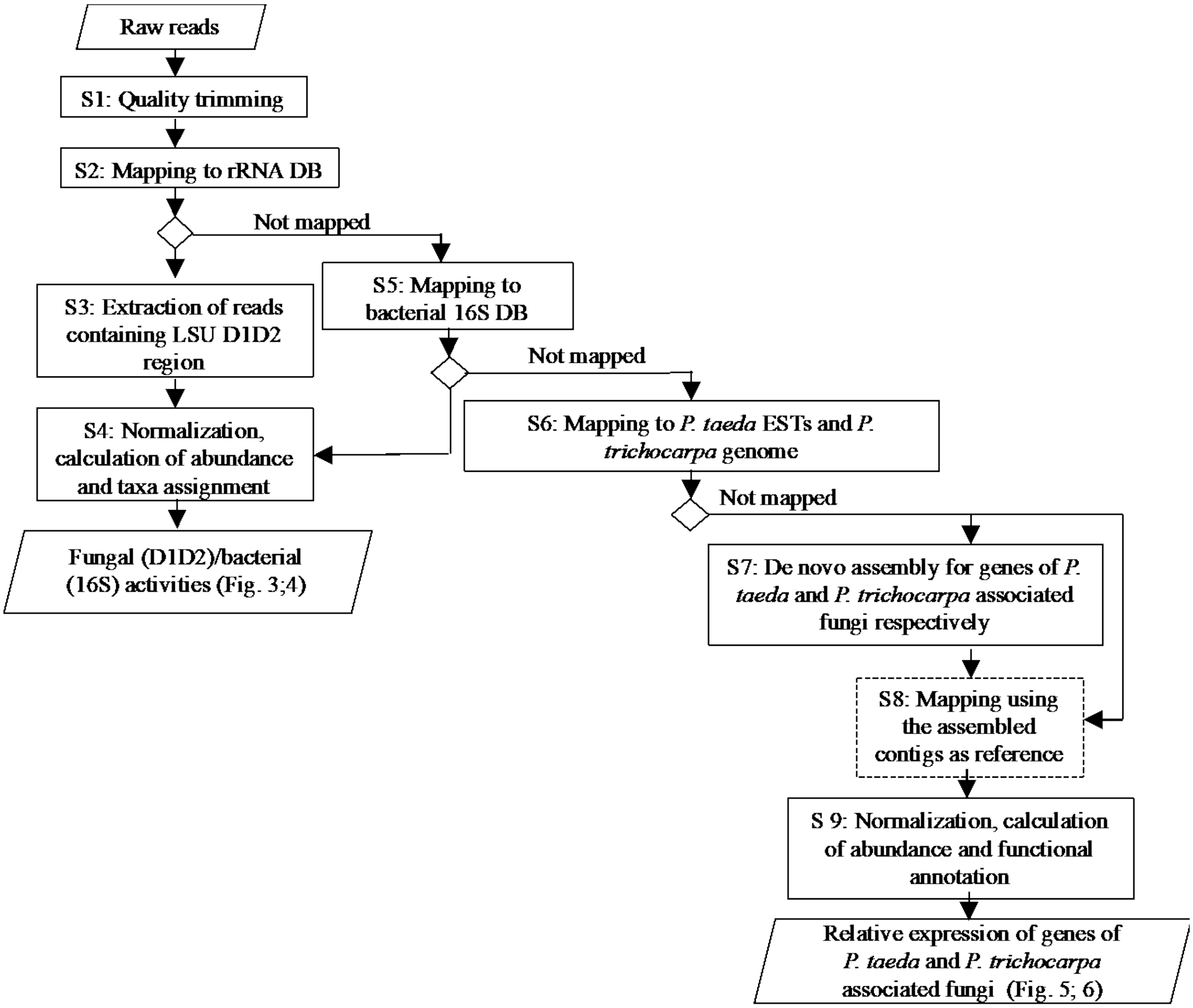

Fig S2. Summary of the computational pipeline used for metatranscriptomic analysis (Step 1 to Step 9, S1-S9). The detailed description for each step was described in SI text A2.

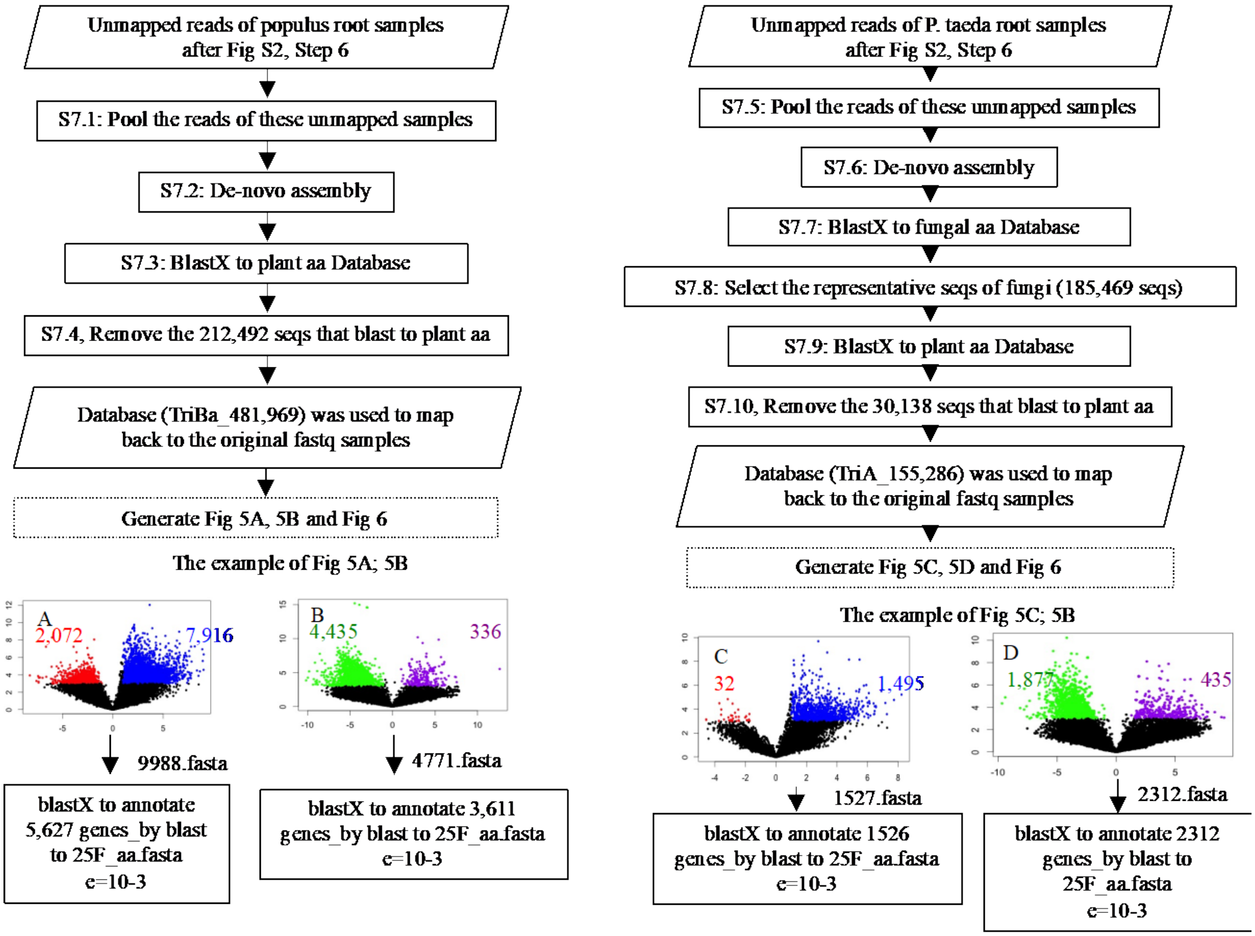

Fig. S3. Sub-workflow showing *de novo* assembly approaches for generating individual databases for genes of **a.** *Populus trichocarpa*-associated fungi (*FunGene_P.tri*) & **b.** *Pinus taeda*-associated fungi (*FunGene_P.taeda*).

Step 7.1: Unmapped reads for the individual *Populus trichocarpa* root samples were collected and pooled, including sample sets: S12_1, S12_2, S12_3, S12_4, S13_5, S13_6, S13_7 (Dataset S1). The pooled sample generated around 26 Gb fastq file for forward and reverse reads respectively.

Step 7.2: *De novo* assembly was applied for the pooled reads with the Trinity package. Representative sequences were extracted to generate 694,333 sequences.

Step 7.3: Sequences generated from Step 7.2 were further filtered using BlastX to the plant amino acid database (*P. taeda* + *P. tri* 210 v3). A total of 212,492 sequences were assigned as plant genes (% ID >50, e<10^-8, alignment length>30).

Step 7.4: Removal of 212,492 plant sequences from *de novo* assembled data (694,333 sequences). Remaining sequences (name: TriBa_481,969.fasta) were used as the "*FunGene_P.tri* database" for mapping to generate Fig. 5A, 5B and Fig. 6.

Step 7.5: Unmapped reads for individual *Pinus taeda* root samples were collected and pooled, including sample sets S13_8, S13_9, S13_10, S13_11, S14_4, S14_5, S14_6 (Dataset S1). Pooled samples generated the fastq files as ~17.3 Gb for forward and reverse reads, respectively.

Step 7.6: *De novo* assembly was applied on pooled reads using the Trinity package. Representative sequences were selected and generated 297,073 sequences.

Step 7.7: Since pine root tips were colonized by a few dominant fungal species (Fig. 3), fungal genome databases were used as references to sort out sequences belonging to the dominate fungi. Here, amino acid sequences from a total of seven fungal genomes (Suihi1, Suidec1, Suigr1, Suibeve1, Suilu1, Rhivi1, Tubme1, 100,930 sequences in total) were used for BlastX (% ID>50, e<=10^-8, alignment length >15).

Step 7.8: Through this approach, 185,469 reprehensive sequences were selected as "fungal genes" from *de novo* assembled sequences (of 297,073 sequences) for the next step.

Step 7.9: Sequences generated from Step 7.8 were further Blasted (BlastX) to plant amino acid databases (*P. taeda* + P. tri 210 v3). A total of 30,138 sequences were blasted as plant genes (% ID >50, e<10^-8, alignment length>30).

Step 7.10: Remove the 30,138 plant sequences from *de novo* assembled data. Remaining sequences (name: TriA 155,286.fasta) were used as "FunGene_*P. taeda* database" for mapping and generated Fig. 5c, 5d and Fig. 6.

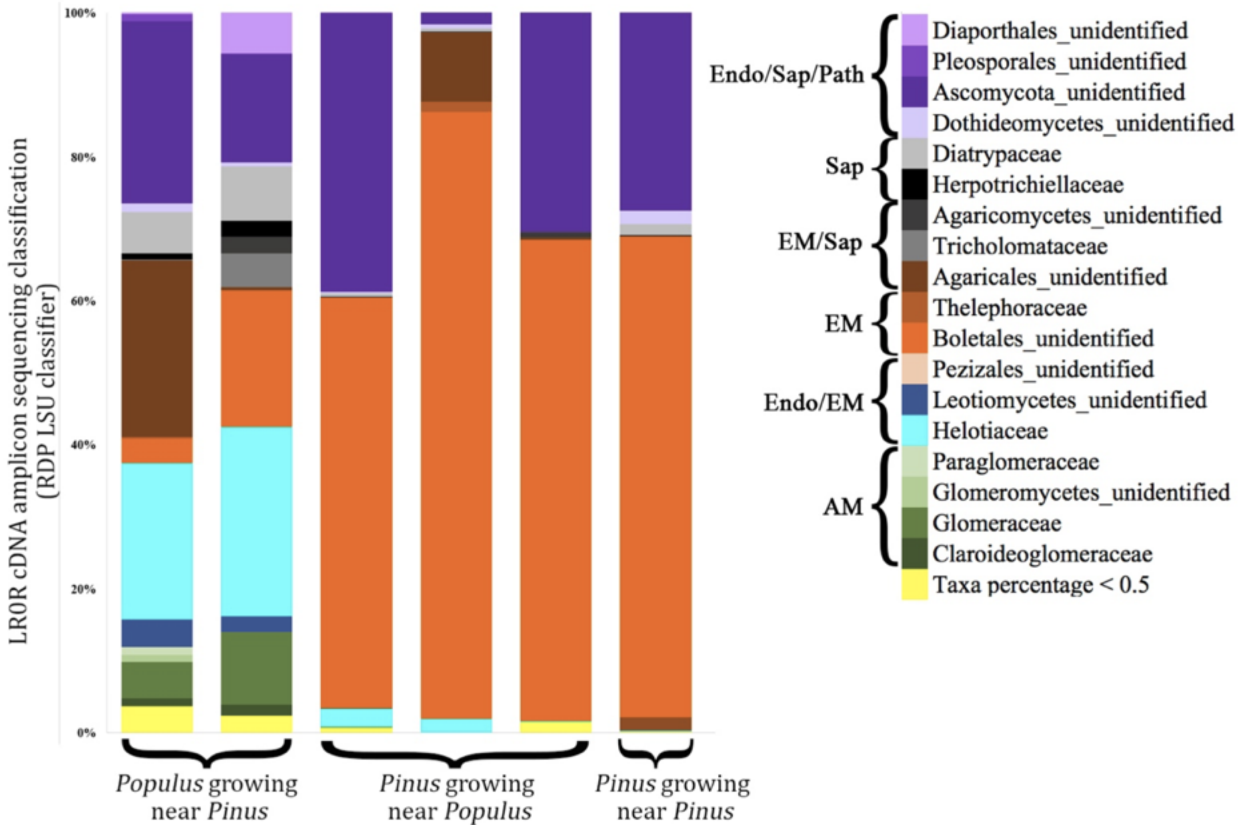

Fig. S4. Transcriptomic abundance (rRNA) of fungal taxa in/on root samples (plant-soil bioassay) based on cDNA amplicon sequencing. Representative sets of the same RNA extraction samples used for RNAseq analysis (shown in Fig. S2, step 3) were used for cDNA amplicon sequencing. The abundances of fungal taxa were determined by read numbers of D1 region of LSU recovered from individual root samples. The color key indicates fungal taxa grouped at the Family level. (classified by RDP classifier with >0.8 bootstrap value). Sequencing preparation and data analysis are shown in SI text A3. (AM, Arbuscular Mycorrhizal Fungi; EM, Ectomycorrhizal Fungi; Sap, Saprotrophic fungi; Endo, Endophytic Fungi; Path, Pathogenic Fungi).

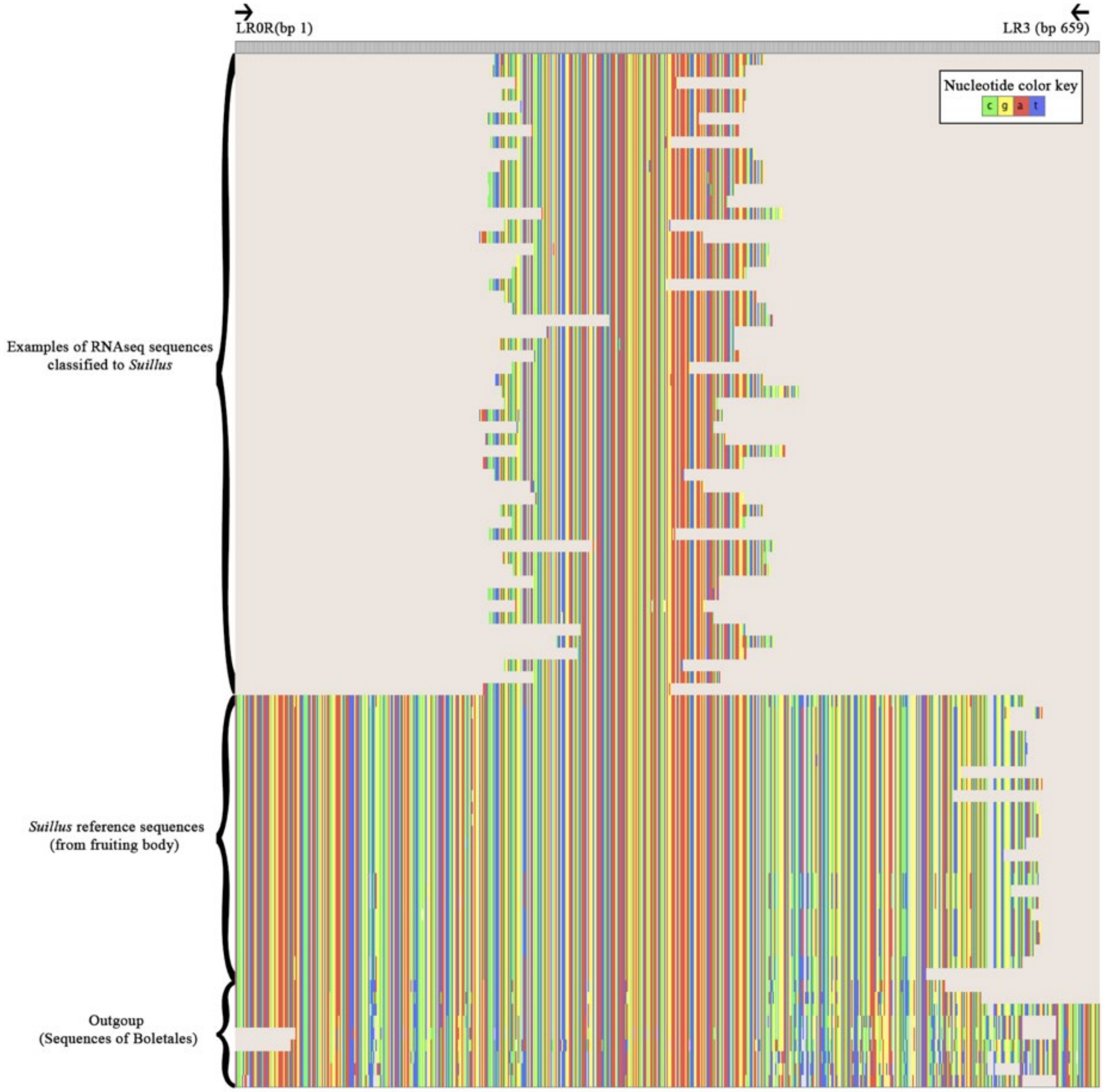

Fig. S5. Schematic figure showing the alignment of D1/D2 regions derived from 1) RNAseq mined *Suillus**, 2) Sanger sequencing of *Suillus* fruiting bodies, and 3) closely-related fungal taxa (Boletales). *Forward and reverse reads of short reads classified to *Suillus* with > 60% bootstrap support (RDP classifier) were merged with USEARCH8. Sequences were aligned with MAFFT and visualized in Mesquite(Maddison & Maddison, 2016). Outgroups (Boletales) included *Xerocomus chrysenteron, Paxillus filamentosus, Coniophora arida, Pseudomerulius*

aureus, Bondarcevomyces taxi, Rhizopogon parksii, Chroogomphus tomentosus, Rhizopogon semireticulatus, Boletus xanthopus.

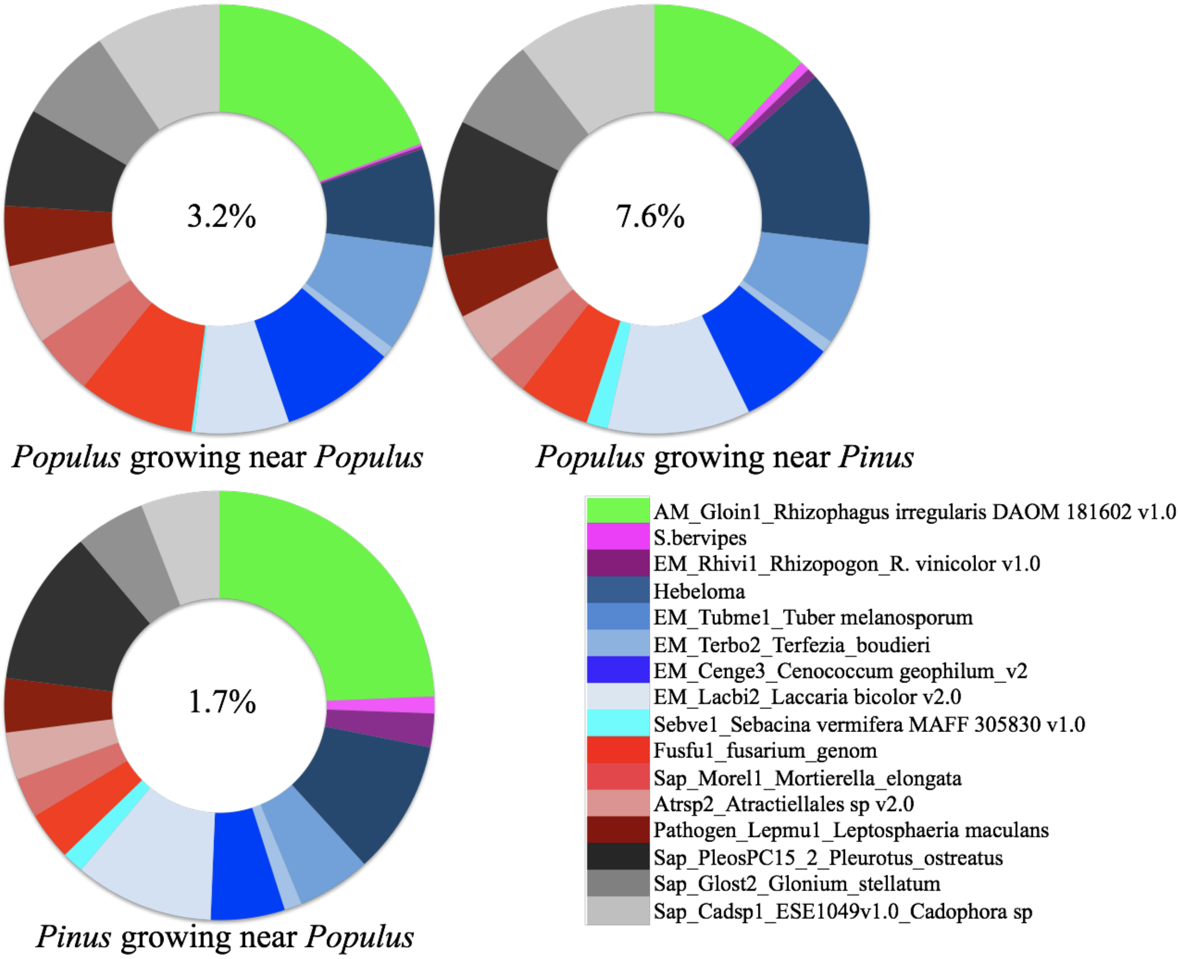

Fig. S6. Percentage (%) of reads recovered from RNAseq of root samples (n=4, in average) that were able to be mapped to publically available fungal genome databases. In total, 24 fungal genomes were used as databases for genome mapping (SI text A2). The figure shows the data from root samples collected from *Populus* grown with *Populus*, *Populus* grown with *Pinus* and *Pinus* grown with *Populus*. In general, only 1.7% to 7.6% of the total reads were mapped to the to the top 16 dominant fungal genomes.

C. Supplementary Table S1 to S2

Table S1. Percentage (%) of EMF mycorrhization rate across *Pinus taeda* and *Populus trichocarpa* combinations.

Bioassay	% of EMF root tips[1]
P. trichocarpa (*P. trichocarpa* x P. *trichocarpa*)	1.3 ± 0.5
P. trichocarpa (*P. taeda* x *P. trichocarpa*)	1.1 ± 0.5
P. taeda (*P. taeda* x *P. trichocarpa*)	81 ± 13
P. taeda (*P. taeda* x *P. taeda*)	83 ± 12

[1]The percentage of EMF root tips was assessed by counting the numbers of roots that showed morphology characteristic of ectomycorrhizas relative to the total number of tips examined (n≥4). 200 root tips were counted for an individual plant samples. Successful EMF symbiosis on *Pinus taeda and Populus trichocarpa* was characterized based upon several morphological features in developing plant roots and root sections, including the observation of the short, swollen root tips, presence of a fungal sheath (mantle) and Hartig-net (Harley & Smith, 1983; Kottke & Oberwinkler, 1986; Smith & Read, 2010) based on microscopic observation of the root in cross-section. Bifurcated root tips and root clusters (with 5-10 root tips) are quite obvious in *P. taeda* mycorrhizae.

Table S2. Total reads and relative ratio of fungal D1/D2 and bacterial 16S recovered from heterospecific and conspecific plant bioassays with *Pinus taeda* and *Populus trichocarpa*.

Bioassay/No. x 1000 reads	D1D2	16S rRNA	D1D2:16S ratio

P. trichocarpa (*P. trichocarpa* x *P. trichocarpa*)	54 ± 13	39 ± 34	1.4
P. trichocarpa (*P. taeda* x *P. trichocarpa*)	162 ± 91	80 ± 42	2
P. taeda (*P. taeda* x *P. trichocarpa*)	16 ± 6	5 ± 2	3.2
P. taeda (*P. taeda xP. taeda*)	8 ± 1	6 ± 1	1.2

Table S3. Taxonomic composition of root microbiomes in associated with neighboring plant species. PERMANOVA was applied to identify the differential abundance of ribosomal RNA sequences (rRNA LSU D1D2 for fungi, 16S rRNA for bacteria) recovered from the roots of individual plants. In particular, The comparisons **(Table S3a and S3c)** or pairwise comparisons **(Table S3b and S3d)** using PERMANOVA were applied to identify the microbial diversity for whole communities. In addition, pairwise comparisons were applied for each listed fungal taxa (Dataset S2b) and bacterial taxa (Dataset S2d) .

In (a and b), The results were used to generate NMDS plots in Figure 4a and 4b

In (c and d), The results were used to generate NMDS plots in Figure 4c and 4d.

Table S3a. PERMANOVA of fungal rRNA LSU, whole community

		Df	Sums_of_Sqs	MeanSqs	F.Model	R2	Pr(>F)
All	site$label	3	2.8069	0.93563	7.8678	0.68211	**0.001*** **
	residuals	11	1.3081	0.11892		0.31789	

	total	14	4.1150			1.00000	

Note: call = adonis (formula = abDist ~ site$label); Permutation = free; Number of permutations: 999; Signif. codes: 0 ‘***’ 0.001 ‘**’ 0.01 ‘*’ 0.05 ‘.’ 0.1 ‘ ‘

Table S3b. PERMANOVA of fungal rRNA LSU, pairwise comparisons

		Df	Sums_of_Sqs	MeanSqs	F.Model	R2	Pr(>F)
(1) P. tri (P. tri x P. tri) vs. P. tri (P. tri x P. taeda)	site$label	1	0.38048	0.38048	2.9198	0.32734	**0.031***
	residuals	6	0.78186	0.13031		0.67266	
	total	7	1.16234			1.00000	
(2) P. tri (P. tri x P. tri) v.s. P. taeda (P. tri x P. taeda)	site$label	1	1.47289	1.47289	15.644	0.72279	**0.026***
	residuals	6	0.56489	0.09415		0.27721	
	total	7	2.03778			1.00000	
(3) P. tri (P. tri x P. tri) vs. P. taeda (P. taeda x P. taeda)	site	1	1.02720	1.02720	5.8913	0.54092	**0.037***
	residuals	5	0.87179	0.17436		0.45908	
	total	6	1.89900			1.0000	
(4) P. tri (P. tri x P. taeda) vs. P. taeda (P. tri x P. taeda)	site$label	1	1.10360	1.10360	15.176	0.71666	**0.031***
	residuals	6	0.43632	0.07272		0.28334	
	total	7	1.53992			1.00000	
(5) P. tri (P. tri	site	1	0.89491	0.89491	6.0205	0.5463	**0.022***

x P. taeda) vs. P. taeda (P. taeda x P. taeda)	residuals	5	0.74322	0.14864		0.4537	
	total	6	1.63814			1.0000	
(6) P. taeda (P. tri x P. taeda) vs. P. taeda (P. taeda x P. taeda)	site	1	0.71327	0.71327	6.7769	0.57544	**0.024***
	residuals	5	0.52625	0.10525		0.42456	
	total	6	1.23953			1.00000	

Note: call = adonis (formula = abDist ~ site$label[index]); Permutation = free; Number of permutations: 999; Signif. codes: 0 '***' 0.001 '**' 0.01 '*' 0.05 '.' 0.1 ' '; P. tri =*P. trichocarpa*

Table S3c. PERMANOVA of bacterial 16S, whole community

		Df	Sums_of_Sqs	MeanSqs	F.Model	R2	Pr(>F)
All	site$label	3	1.10084	0.36695	11.955	0.76529	**0.001*****
	residuals	11	0.33763	0.03069		0.23471	
	total	14	1.43847			1.00000	

Note: call = adonis (formula = abDist ~ site$label); Permutation = free; Number of permutations: 999; Signif. codes: 0 '***' 0.001 '**' 0.01 '*' 0.05 '.' 0.1 ' '

Table S3d. PERMANOVA of bacterial 16S, pairwise comparisons

		Df	Sums_of_Sqs	MeanSqs	F.Model	R2	Pr(>F)

(1) P. tri (P. tri x P. tri) vs. P. tri (P. tri x P. taeda)	site$label	1	0.039449	0.039449	1.3606	0.18485	**0.192**
	residuals	6	0.173961	0.028993		0.81515	
	total	7	0.213410			1.00000	
(2) P. tri (P. tri x P. tri) vs. P. taeda (P. tri x P. taeda)	site$label	1	0.32069	0.32069	7.1346	0.54319	**0.034***
	residuals	6	0.26969	0.04495		0.45681	
	total	7	0.59038			1.00000	
(3) P. tri (P. tri x P. tri) vs. P. taeda (P. taeda x P. taeda)	site	1	0.48960	0.4896	21.758	0.81314	**0.028***
	residuals	5	0.11251	0.0225		0.18686	
	total	6	0.60211			1.00000	
(4) P. tri (P. tri x P. taeda) vs. P. taeda (P. tri x P. taeda)	site$label	1	0.20599	0.20599	5.4901	0.47781	**0.021***
	residuals	6	0.22512	0.03752		0.52219	
	total	7	0.43111			1.00000	
(5) P. tri (P. tri x P. taeda) vs. P. taeda (P. taeda x P. taeda)	site	1	0.50562	0.50562	37.213	0.88155	**0.03***
	residuals	5	0.06794	0.01359		0.11845	
	total	6	0.57355			1.00000	
(6) P. taeda (P. tri x P. taeda) vs. P. taeda (P. taeda x P. taeda)	site	1	0.71673	0.71673	21.896	0.8141	**0.027***
	residuals	5	0.16367	0.03273		0.1859	
	total	6	0.88040			1.00000	

Note: call = adonis (formula = abDist ~ site$label[index]); Permutation = free; Number of permutations: 999; Signif. codes: 0 ‘***’ 0.001 ‘**’ 0.01 ‘*’ 0.05 ‘.’ 0.1 ‘ ‘

References

Alfaro M, Castanera R, Lavín JL, Grigoriev IV, Oguiza JA, Ramírez L, Pisabarro AG. (**2016**) Comparative and transcriptional analysis of the predicted secretome in the lignocellulose-degrading basidiomycete fungus Pleurotus ostreatus. *Environmental microbiology* **18**, 4710–4726.

Blankenberg D, Gordon A, Von Kuster G, Coraor N, Taylor J, Nekrutenko A, Galaxy Team. (**2010**) Manipulation of FASTQ data with Galaxy. *Bioinformatics* **26**, 1783–1785.

Bonito G, Hameed K, Ventura R, Krishnan J, Schadt CW, Vilgalys R. (**2016**) Isolating a functionally relevant guild of fungi from the root microbiome of Populus. *Fungal ecology* **22**, 35–42.

Branco S, Gladieux P, Ellison CE, Kuo A, LaButti K, Lipzen A, Grigoriev IV, Liao H-L, Vilgalys R, Peay KG, *et al.* (**2015**) Genetic isolation between two recently diverged populations of a symbiotic fungus. *Molecular ecology* **24**, 2747–2758.

Caporaso JG, Kuczynski J, Stombaugh J, Bittinger K, Bushman FD, Costello EK, Fierer N, Peña AG, Goodrich JK, Gordon JI, *et al.* (**2010**) QIIME allows analysis of high-throughput community sequencing data. *Nature methods* **7**, 335–336.

Castanera R, López-Varas L, Borgognone A, LaButti K, Lapidus A, Schmutz J, Grimwood J, Pérez G, Pisabarro AG, Grigoriev IV, *et al.* (**2016**) Transposable Elements versus the Fungal Genome: Impact on Whole-Genome Architecture and Transcriptional Profiles. *PLoS genetics* **12**, e1006108.

Clarke KR, Somerfield PJ, Chapman MG. (**2006**) On resemblance measures for ecological studies, including taxonomic dissimilarities and a zero-adjusted Bray–Curtis coefficient for denuded assemblages. *Journal of experimental marine biology and ecology* **330**, 55–80.

Cole JR, Wang Q, Fish JA, Chai B, McGarrell DM, Sun Y, Brown CT, Porras-Alfaro A, Kuske CR, Tiedje JM. (**2014**) Ribosomal Database Project: data and tools for high throughput rRNA analysis. *Nucleic acids research* **42**, D633–42.

Cuomo CA, Güldener U, Xu J-R, Trail F, Turgeon BG, Di Pietro A, Walton JD, Ma L-J, Baker SE, Rep M, *et al.* (**2007**) The Fusarium graminearum genome reveals a link between localized polymorphism and pathogen specialization. *Science* **317**, 1400–1402.

Deshpande V, Wang Q, Greenfield P, Charleston M, Porras-Alfaro A, Kuske CR, Cole JR, Midgley DJ, Tran-Dinh N. (**2016**) Fungal identification using a Bayesian classifier and the

Warcup training set of internal transcribed spacer sequences. *Mycologia* **108**, 1–5.

Edgar RC. (**2013**) UPARSE: highly accurate OTU sequences from microbial amplicon reads. *Nature methods* **10**, 996–998.

Harley JL, Smith SE. (**1983**) *Mycorrhizal symbiosis*. Academic Press. London, 483.

Johansen RB, Johnston P, Mieczkowski P, Perry GLW, Robeson MS, Burns BR, Vilgalys R. (**2016**) A native and an invasive dune grass share similar, patchily distributed, root-associated fungal communities. *Fungal ecology* **23**, 141–155.

Knapp DG, Németh JB, Barry K, Hainaut M, Henrissat B, Johnson J, Kuo A, Lim JHP, Lipzen A, Nolan M, *et al.* (**2018**) Comparative genomics provides insights into the lifestyle and reveals functional heterogeneity of dark septate endophytic fungi. *Scientific reports* **8**, 6321.

Kohler A, Kuo A, Nagy LG, Morin E, Barry KW, Buscot F, Canbäck B, Choi C, Cichocki N, Clum A, *et al.* (**2015**) Convergent losses of decay mechanisms and rapid turnover of symbiosis genes in mycorrhizal mutualists. *Nature genetics* **47**, 410–415.

Kottke I, Oberwinkler F. (**1986**) Mycorrhiza of forest trees—structure and function. *Trees-Structure and Function* **1**, 1–24.

Langmead B, Salzberg SL. (**2012**) Fast gapped-read alignment with Bowtie 2. *Nature methods* **9**, 357–359.

Liao H-L, Chen Y, Bruns TD, Peay KG, Taylor JW, Branco S, Talbot JM, Vilgalys R. (**2014**) Metatranscriptomic analysis of ectomycorrhizal roots reveals genes associated with Piloderma-Pinus symbiosis: improved methodologies for assessing gene expression in situ. *Environmental microbiology* **16**, 3730–3742.

Liao H-L, Chen Y, Vilgalys R. (**2016**) Metatranscriptomic study of common and host-specific patterns of gene expression between pines and their symbiotic ectomycorrhizal fungi in the genus Suillus. *PLoS genetics* **12**, e1006348.

Li H, Handsaker B, Wysoker A, Fennell T, Ruan J, Homer N, Marth G, Abecasis G, Durbin R, 1000 Genome Project Data Processing Subgroup. (**2009**) The Sequence Alignment/Map format and SAMtools. *Bioinformatics* **25**, 2078–2079.

Maddison WP, Maddison DR. (**2016**) Mesquite: a modular system for evolutionary analysis. Version 3.04. 2015.

Martin M. (**2011**) Cutadapt removes adapter sequences from high-throughput sequencing reads. *EMBnet.journal* **17**, 10–12.

Martin F, Aerts A, Ahrén D, Brun A, Danchin EGJ, Duchaussoy F, Gibon J, Kohler A, Lindquist E, Pereda V, *et al.* (**2008**) The genome of Laccaria bicolor provides insights into mycorrhizal symbiosis. *Nature* **452**, 88–92.

Martin F, Kohler A, Murat C, Balestrini R, Coutinho PM, Jaillon O, Montanini B, Morin E, Noel B, Percudani R, *et al.* (2010) Périgord black truffle genome uncovers evolutionary origins and mechanisms of symbiosis. *Nature* **464**, 1033–1038.

Mujic AB, Kuo A, Tritt A, Lipzen A, Chen C, Johnson J, Sharma A, Barry K, Grigoriev IV, Spatafora JW. (**2017**) Comparative Genomics of the Ectomycorrhizal Sister Species Rhizopogon vinicolor and Rhizopogon vesiculosus (Basidiomycota: Boletales) Reveals a Divergence of the Mating Type B Locus. *G3: Genes, Genomes, Genetics* **7**, 1775–1789.

Nguyen, N. H., Song, Z., Bates, S. T., Branco, S., Tedersoo, L., Menke, J., et al. (2016). FUNGuild: An open annotation tool for parsing fungal community datasets by ecological guild. Fungal Ecol. 20, 241–248.

Peter M, Kohler A, Ohm RA, Kuo A, Krützmann J, Morin E, Arend M, Barry KW, Binder M, Choi C, *et al.* (**2016**) Ectomycorrhizal ecology is imprinted in the genome of the dominant symbiotic fungus Cenococcum geophilum. *Nature communications* **7**, 12662.

R Development Core Team. (**2003**) *The R Reference Manual: Base Package*. Network Theory.

Riley R, Salamov AA, Brown DW, Nagy LG, Floudas D, Held BW, Levasseur A, Lombard V, Morin E, Otillar R, *et al.* (**2014**) Extensive sampling of basidiomycete genomes demonstrates inadequacy of the white-rot/brown-rot paradigm for wood decay fungi (Proceedings of the National Academy of Sciences of the United States of America (2014) 111, 27 (9923-9928. *Proceedings of the National Academy of Sciences of the United States of America* **111**, 14959.

Rouxel T, Grandaubert J, Hane JK, Hoede C, van de Wouw AP, Couloux A, Dominguez V, Anthouard V, Bally P, Bourras S, *et al.* (**2011**) Effector diversification within compartments of the Leptosphaeria maculans genome affected by Repeat-Induced Point mutations. *Nature communications* **2**, 202.

Smith SE, Read DJ. **2010**. *Mycorrhizal Symbiosis*. Academic Press.

Tisserant E, Malbreil M, Kuo A, Kohler A, Symeonidi A, Balestrini R, Charron P, Duensing N, Frei dit Frey N, Gianinazzi-Pearson V, *et al.* (**2013**) Genome of an arbuscular mycorrhizal fungus provides insight into the oldest plant symbiosis. *Proceedings of the National Academy of Sciences of the United States of America* **110**, 20117–20122.

Trapnell C, Pachter L, Salzberg SL. (**2009**) TopHat: discovering splice junctions with RNA-Seq. *Bioinformatics* **25**, 1105–1111.

Tuskan GA, Difazio S, Jansson S, Bohlmann J, Grigoriev I, Hellsten U, Putnam N, Ralph S, Rombauts S, Salamov A, *et al.* (**2006**) The genome of black cottonwood, Populus trichocarpa (Torr. & Gray). *Science* **313**, 1596–1604.

Uehling J, Gryganskyi A, Hameed K, Tschaplinski T, Misztal PK, Wu S, Desirò A, Vande Pol N, Du Z, Zienkiewicz A, *et al.* (**2017**) Comparative genomics of *Mortierella elongata* and its bacterial endosymbiont Mycoavidus cysteinexigens. *Environmental microbiology* **19**, 2964–

2983.

Vilgalys R, Hester M. (**1990**) Rapid genetic identification and mapping of enzymatically amplified ribosomal DNA from several Cryptococcus species. *Journal of bacteriology* **172**, 4238–4246.
